# Evolution of sexually dimorphic pheromone profiles coincides with increased number of male‐specific chemosensory organs in *Drosophila prolongata*

**DOI:** 10.1002/ece3.5819

**Published:** 2019-11-17

**Authors:** Yige Luo, Yunwei Zhang, Jean‐Pierre Farine, Jean‐François Ferveur, Santiago Ramírez, Artyom Kopp

**Affiliations:** ^1^ Department of Evolution and Ecology University of California‐Davis Davis CA USA; ^2^ Department of Statistics University of California‐Davis Davis CA USA; ^3^ Centre des Sciences du Goût et de l'Alimentation Université de Bourgogne‐Dijon Dijon France; ^4^ Present address: School of Mathematics and Statistics University of Sydney Sydney NSW Australia

**Keywords:** cuticular hydrocarbons, pheromones, sex‐limited polymorphism, sexual dimorphism

## Abstract

Binary communication systems that involve sex‐specific signaling and sex‐specific signal perception play a key role in sexual selection and in the evolution of sexually dimorphic traits. The driving forces and genetic changes underlying such traits can be investigated in systems where sex‐specific signaling and perception have emerged recently and show evidence of potential coevolution. A promising model is found in *Drosophila prolongata*, which exhibits a species‐specific increase in the number of male chemosensory bristles. We show that this transition coincides with recent evolutionary changes in cuticular hydrocarbon (CHC) profiles. Long‐chain CHCs that are sexually monomorphic in the closest relatives of *D. prolongata* (*D. rhopaloa*, *D. carrolli*, *D. kurseongensis*, and *D. fuyamai*) are strongly male‐biased in this species. We also identify an intraspecific female‐limited polymorphism, where some females have male‐like CHC profiles. Both the origin of sexually dimorphic CHC profiles and the female‐limited polymorphism in *D. prolongata* involve changes in the relative amounts of three mono‐alkene homologs, 9‐tricosene, 9‐pentacosene, and 9‐heptacosene, all of which share a common biosynthetic origin and point to a potentially simple genetic change underlying these traits. Our results suggest that pheromone synthesis may have coevolved with chemosensory perception and open the way for reconstructing the origin of sexual dimorphism in this communication system.

## Graphical abstract

Uniquely among *Drosophila* species, *Drosophila prolongata* shows a dramatic male‐specific expansion of the leg chemosensory organs. We show that this change is accompanied by an equally recent evolution of sexually dimorphic pheromones. Long‐chain hydrocarbons that are sexually monomorphic in other species are strongly enriched in *D. prolongata* males.

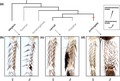

## 1 INTRODUCTION

Evolutionary biologists have long recognized that sexual selection plays a major role in the evolution and diversification of sexually dimorphic traits (Andersson, 1994; Andersson & Simmons, 2006; Darwin, 1871; Lande, 1980; Lande & Arnold, 1985). In animals, communication between and within the sexes can lead to sexual selection and result in the evolution of sexual dimorphism (Andersson, 1994). Several mechanisms have been proposed to explain the emergence of sexually dimorphic traits via sexual selection. The indicator mechanism (Williams, 1966) and the sensory exploitation hypothesis (Ryan, 1990) focus on the directional selection exerted by one the signaling mechanism (emitter component). The Fisherian runaway model (Fisher, 1930) emphasizes the existence of a genetic correlation between the emitter and receiver components, leading to a phenomenon of reciprocal and self‐reinforcing selection between signaling trait(s) and signal preference(s). These mechanisms often work in concert, and as a result, correlated evolutionary changes are generally expected between the expression and perception of secondary sexual traits (Andersson, 1994). For example, in three‐spined stickleback, males with the brightest red breeding coloration are preferred by females (Milinski & Bakker, 1990), whose visual sensitivity in the red spectrum increases during the reproductive season (Cronly‐Dillon & Sharma, 1968).

Insects rely on chemical communication to locate, identify, and select mates. Chemical cues and their corresponding receptors offer an excellent opportunity to study the evolution of sexual dimorphism (Steiger & Stökl, 2014). Insect pheromones, which include both volatile and contact (cuticular) hydrocarbons (CHCs), are critical for courtship and mating behavior (Blomquist & Bagnères, 2010; Stanley & Nelson, 1993). Sexually dimorphic pheromones have been discovered across many insect taxa (Howard & Blomquist, 2005). Similar to the pattern often observed in visual communication systems, sex‐specific pheromones have coevolved with their cognate receptors. Many examples of correlated evolutionary changes between CHC production and perception have been documented, with evolutionary gains and losses of sex‐specific CHCs mirroring evolutionary gains and losses of sensory response to these CHCs (Choe & Crespi, 1997; Dekker et al., 2015; Ng et al., 2014; Sappington & Taylor, 1990).

The genetic mechanisms that control pheromone production and perception are best understood in *Drosophila melanogaster* (Ferveur, 2005). *Drosophila* pheromones are produced in specialized oenocyte cells in a chain of chemical reactions catalyzed by fatty acyl synthases, desaturases, elongases, and reductases (Ferveur et al., 1997; Howard & Blomquist, 1982; Jallon, 1984; Wicker‐Thomas et al., 2015). At the same time, more than 150 chemoreceptor proteins have been identified, including the odorant receptor (OR) and gustatory receptor (GR) families, the ionotropic receptor (IR) family, pickpocket (ppk) channels, and some transient receptor potential (TRP) channels (Depetris‐Chauvin, Galagovsky, & Grosjean, 2015). One of the best studied examples is the female‐specific courtship‐inducing diene pheromone of *D. melanogaster*, 7,11‐heptacosadiene (7,11‐HD; Antony & Jallon, 1982). 7,11‐HD is produced by female‐biased expression of a desaturase (*desatF*) and an elongase (*eloF*; Chertemps et al., 2007; Chertemps, Duportets, Labeur, Ueyama, & Wicker‐Thomas, 2006), while its cognate receptor *ppk23* is expressed in males (Toda, Zhao, & Dickson, 2012). Remarkably, in *D. simulans*, the closest relative of *D. melanogaster*, male courtship is instead strongly inhibited by 7,11‐HD (Marcillac, Houot, & Ferveur, 2005; Savarit, Sureau, Cobb, & Ferveur, 1999). This difference appears to be due not to the peripheral neurons that express *ppk23*, but rather to interspecific differences in the processing of sensory information in the brain (Seeholzer, Seppo, Stern, & Ruta, 2018).

The best models for understanding evolutionary innovations in male–female communication are those where sexual dimorphism in both signaling and perception has evolved recently from an ancestral monomorphic state. However, among the closest relatives of *D. melanogaster* (the *melanogaster* species subgroup), sexually dimorphic expression of 7,11‐HD appears to be ancestral, with most evolutionary changes due to secondary losses of sex‐biased dienes (Jallon & David, 1987; Shirangi, Dufour, Williams, & Carroll, 2009). The genus *Drosophila*, with >1,600 described species (O'Grady & DeSalle, 2018), is likely to harbor a much greater diversity of sex‐specific CHC profiles. Most of this diversity remains unexplored (Ferveur, 2005).

**Figure 1:**
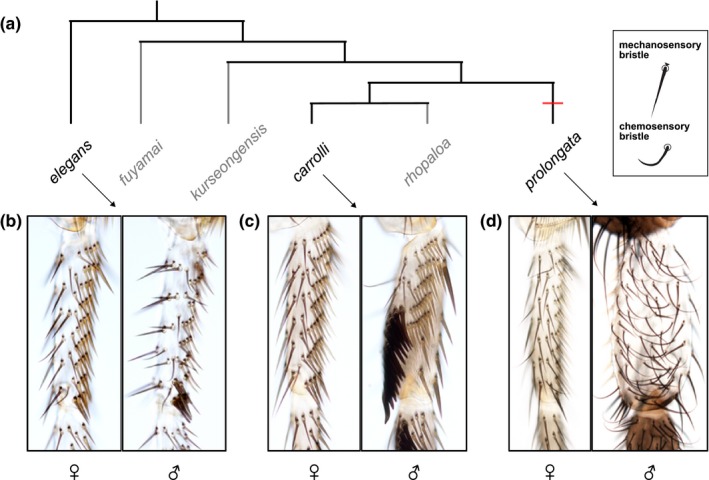
Evolution of a sexually dimorphic chemosensory system in *Drosophila prolongata*. (a) Phylogenetic relationships among the species used in this study (Based on Barmina & Kopp, 2007). *Drosophila elegans* is included as an outgroup to the *rhopaloa* species subgroup, which encompasses the remaining species. Species for which leg images are shown are indicated in black, the rest are in light gray. Red bar indicates the inferred timing of male‐specific expansion of foreleg gustatory organs. (b–d) Proximal foreleg tarsi of *D. elegans* (b), *D. carrolli *(c), and *D. prolongata* (d), with females on the left and males on the right. The two types of sensory organs can be distinguished by external morphology: mechanosensory bristles are straight, pointed, and bear triangular bracts at the base, whereas gustatory bristles are curved, bractless, and have rounded tips. Note the dramatic increase in the number of gustatory organs in *D. prolongata* males

*Drosophila prolongata*, a member of the *melanogaster* species group (Singh & Gupta, 1977; Toda, 1991), is a promising model to study both the selective forces and the genetic mechanisms behind sexual dimorphism. This species displays several striking sexually dimorphic traits, including the exaggerated male forelegs that distinguish *D. prolongata* from closely related species (Singh & Gupta, 1977) and are essential for courtship and mating success (Setoguchi et al., 2014). This sex‐specific increase in leg size has been accompanied by an equally recent male‐specific expansion of the chemosensory system (Figure 1, see Section 3). Because foreleg chemoreceptors are directly involved in pheromone perception (Fan et al., 2013; Inoshita, Martin, Marion‐Poll, & Ferveur, 2011; Stocker, 1994), we hypothesized that pheromone profiles may also display a strong degree of sexual dimorphism in *D. prolongata*. To test this hypothesis, we characterized the male and female CHC profiles of *D. prolongata* and its relatives. We found that, indeed, the CHC profiles of *D. prolongata* are highly distinct from all other species, due primarily to a strong male‐specific increase in the amount of long‐chain CHCs. We also observe an intriguing female‐limited CHC polymorphism in this species, where some females have male‐like CHC profiles. These results set the stage for investigating potential coevolution between sex‐specific pheromone production and perception on a recent evolutionary time scale.

## 2 MATERIALS AND METHODS

### 2.1 Fly stocks

The species included in this study, *D. prolongata* (Singh & Gupta, 1977), *D. carrolli* (Gompel & Kopp, 2018), *D. rhopaloa* (Bock & Wheeler, 1972), *D. kurseongensis* (Gupta & Singh, 1977), and *D. fuyamai* (Toda, 1991), are members of the *rhopaloa* species subgroup (Toda, 1991), a well‐supported monophyletic clade (Barmina & Kopp, 2007). For each species, we used isofemale strains that were derived from a single wild‐caught female and maintained in the laboratory for many generations. Two strains of *D. prolongata*, referred to as *D. prolongata* Sapa and *D. prolongata* Bavi hereafter, *D. rhopaloa* (strain BaVi067), and *D. kurseongensis* (SaPa058), were originally collected by H. Takamori in Vietnam in September 2004, March 2005, September 2004, and March 2009, respectively. *D. carrolli* (KB866) and *D. fuyamai* (KB1217) were collected by A. Kopp and O. Barmina at Kuala Belalong, Brunei in October 2003. For all strains, cultures of around 150 flies each were raised in bottles on standard cornmeal media at room temperature, approximately 40% humidity, under natural light cycle regime. Within 6 hr after emergence, virgin flies were sexed, isolated, and kept singly in individual vials with the same media. All flies were aged for 7 days before cuticular hydrocarbon extraction.

### 2.2 Specimen preparation and microscopic imaging

Prothoracic legs were dissected from adult female and male flies under CO_2_ anesthesia and mounted in Hoyer's media between two coverslips. After overnight clearing, the legs were imaged under bright field illumination with a 20× lens on a Leica DM500B microscope with a Leica DC500 camera. Stacks of images were merged into single extended depth‐of‐field images and processed further using Adobe Photoshop.

### 2.3 Cuticular hydrocarbon (CHC) extraction

After freezing at −20°C for 15 min, flies were individually plunged into hexane‐containing glass inserts (Agilent Technologies) seated in 2‐ml vials (Agilent Technologies). During processing, flies were handled with acetone‐washed titanium forceps to prevent cross‐contamination of cuticular hydrocarbons. Flies were soaked in 30‐μl pure hexane for 5 min, followed by 30 s gentle vortexing. After the fly was removed, each extraction vial was left open for 2 hr to desiccate. All samples were sealed with Teflon caps (Agilent Technologies) and stored at −20°C. Prior to analysis, samples were resolubilized by adding 15 μl hexane with two spiked‐in alkanes: n‐hexacosane (Sigma‐Aldrich) and n‐triacotane (Sigma‐Aldrich), 10 ng/μl each. These compounds were absent in both sexes across all studied species and thus were selected as external standards, referred to as ES‐1 and ES‐2 hereafter.

### 2.4 Gas chromatography (GC) and mass spectrometry (MS) analyses

The cuticular hydrocarbon extracts were analyzed on an Agilent 7890B GC fitted with a 30 m × 0.25 mm × 0.25 μm HP‐5 Ultra Inert column and coupled to an Agilent 5977A mass spectrometer (Agilent Technologies). One microliter sample was introduced to the injection port using an Agilent 7683B autosampler in split‐less mode. The oven temperature was programmed as follows: ramped from 160 to 280°C at a rate of 2.5°C/min, held at 280°C for 1 min, and increased to 315°C at 15°C/min followed by 1 min final hold. The injector and transfer line temperature were kept constant at 275 and 280°C, respectively. Helium was used as the carrier gas with a constant flow rate at 2 ml/min.

The mass selective detector (MSD) was operated to have a 70 eV‐energized electron flow during electron impact ionization, with default temperature settings (ion source at 150°C, quadrupole at 230°C). Mass spectrum was constructed once per 0.188 s (corresponding to a scan rate of 5.31 scans/s), by histogramming ions detected in the range between 30 and 550 m/z.

All GC‐MS data were analyzed using MSD ChemStation Enhanced Data Analysis Software vF.01.00 (Agilent). Putative structures of analytes were inferred by comparing fragmentation patterns to those in the NIST 05 reference library and those in previous *Drosophila* pheromone publications (Dembeck et al., 2015; Everaerts, Farine, Cobb, & Ferveur, 2010; Howard, Jackson, Banse, & Blows, 2003). DMDS‐derivatization reactions were not conducted to confirm the position of the double bonds.

**Figure 2:**
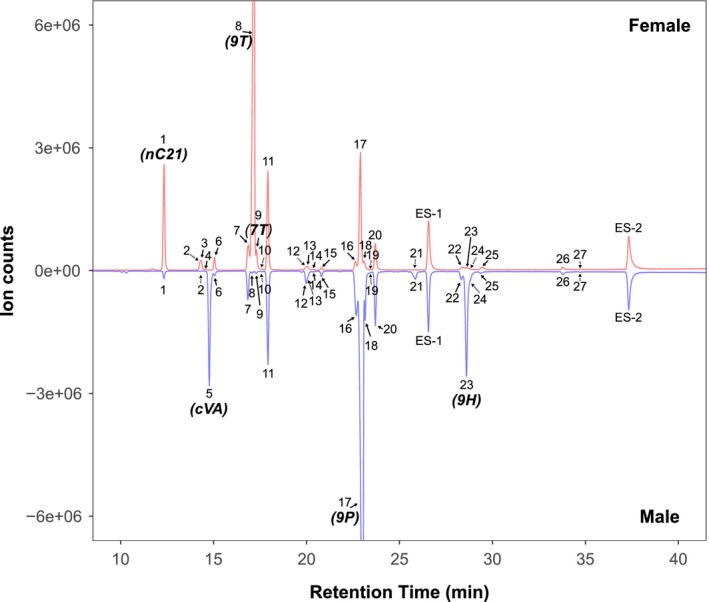
Sexually dimorphic CHC profiles in *Drosophila prolongata*. The graphs show representative GC‐MS chromatograms of cuticular CHCs of a single 7‐day‐old virgin male and female from the Sapa strain, with the female at the top (red) and male in mirror image at the bottom (blue). Compounds corresponding to each numbered peak are listed in Table 1. Compounds that are shared between sexes bear the same number. Arrows indicate peaks that were not perfectly resolved, with the minor component shown by dashed lines. Unit‐less abundances are direct measurements from the mass selective detector. *ES‐1* (n‐hexacosane) and *ES‐2* (n‐triacotane) are external standards used to calculate the absolute amounts of each compound in females (1,953 ± 164 ng; *n * = 24) and males (3,348 ± 263 ng; *n * = 22). Sexually dimorphic compounds include the female‐biased n‐heneicosane (nC21), 9‐tricosene (9T), and 7‐tricosene (7T) and the male‐biased 9‐pentacosene (9P) and 9‐heptacosene (9H)

Representative CHC profiles in both sexes of all studied species are presented in Figure 2 and Figure S1 (also see nomenclature in Table 1). For quantification, individual chromatographic peaks were first called using the built‐in ChemStation integrator with initial peak width 0.045 and initial threshold 16, followed by manual adjustment to include minor peaks and deconvolute overlapping peaks. Consensus peaks were first constructed within groups defined by species and sex, by aligning orthologous peaks among biological replicates using retention time. The final consensus was obtained by merging group consensuses based on inferred chemical identities and/or Kovats indices (KI; Carlson, Bernier, & Sutton, 1998). Cuticular hydrocarbons were initially quantified by measuring individual peak areas and then scaled by external standards to obtain absolute amounts (in nanograms, summarized in Table S2) using the R Studio software (R Core Team, 2018). Analytes with KI <2,600 and >2,600 were scaled by ES‐1 and ES‐2, respectively. Response factors were not determined for individual CHC‐containing peaks.

**Table 1:** Complete list of CHCs identified by GC‐MS

No^a^	Compound name	Abbreviation	Chain length	Chemical class	Characteristic Ions (m/z)	Kovats index
1	n‐Heneicosane	nC21	21	Linear alkane	296	2100
2	9‐Docosene	9D	22	9‐Monoene	308	2172
3	7‐Docosene	7D	22	7‐Monoene	308	2177
4	5‐Docosene	5D	22	5‐Monoene	308	2182
5	11‐cis‐Vaccenyl Acetate	cVA	20	Acetate ester	250, 310	2190
6	n‐Docosane	nC22	22	Linear alkane	310	2200
7	2‐Methyl‐Docosane	23Br	23	Methyl‐branched alkane	281, 309, 324	2263
8	9‐Tricosene	9T	23	9‐Monoene	322	2273
9	7‐Tricosene	7T	23	7‐Monoene	322	2279
10	5‐Tricosene	5T	23	5‐Monoene	322	2288
11	n‐Tricosane	nC23	23	Linear alkane	324	2300
12	9‐Tetracosene	9Te	24	9‐Monoene	336	2372
13	7‐Tetracosene	7Te	24	7‐Monoene	336	2378
14	5‐Tetracosene	5Te	24	5‐Monoene	336	2384
15	n‐Tetracosane	nC24	25	Linear alkane	338	2400
16	2‐Methyl‐Tetracosane	25Br	25	Methyl‐branched alkane	309, 337, 352	2464
17	9‐Pentacosene	9P	25	9‐Monoene	350	2473
18	7‐Pentacosene	7P	25	7‐Monoene	350	2480
19	5‐Pentacosene	5P	25	5‐Monoene	350	2489
20	n‐Pentacosane	nC25	25	Linear alkane	352	2500
21	9‐Hexacosene	9He	26	9‐Monoene	364	2575
22	2‐Methyl‐Hexacosane	27Br	27	Methyl‐branched alkane	337, 365, 380	2663
23	9‐Heptacosene	9H	27	9‐Monoene	378	2672
24	7‐Heptacosene	7H	27	7‐Monoene	378	2678
25	n‐Heptacosane	nC27	27	Linear alkane	380	2700
26	2‐Methyl‐Octacosane	29Br	29	Methyl‐branched alkane	365, 393, 408	2864
27	n‐Nonacosane	nC29	29	Linear alkane	408	2900

a Peak numbers corresponded to the elution order of each analyte and to the peaks shown in Figure 1 and Figure S1.

### 2.5 Statistical analysis

Both univariate and multivariate statistical approaches were implemented to investigate the variation in CHC profiles between sexes and across species and strains. To reduce the effects caused by variation in the absolute amounts of CHCs due to body size differences, proportions of each CHC to the total CHC blends were used during univariate pairwise comparisons. Briefly, each compound was normalized by dividing its absolute amount by the total absolute amount of all components. The non‐CHC component cVA was excluded as it is produced in the ejaculatory bulb instead of oenocytes (Chertemps, Duportets, Labeur, & Wicker‐Thomas, 2005; Guiraudie‐Capraz, Pho, & Jallon, 2007).

For multivariate analysis, proportions of each compound were square root transformed and centered to have zero means, but not scaled to have unit variance. The transformation reduced bias against minor compounds and relaxed the unit‐sum constraint within sample, that is compound proportions add up to 1. Principal component analysis (PCA) was performed on the variance–covariance matrix of the transformed CHC compositions using the function “prcomp” in the “stats” package (R Core Team, 2018). This method visually maximized variation among individuals in reduced space dimensions, with individuals sharing similar CHC compositions clustered together. PCA was also performed on log transformed data (Figure S3A) and on the data that included cVA (Figure S3B). In addition to PCA, nonmetric multidimensional scaling (nMDS) analysis was conducted as an alternative noneigenvector approach to examine the spatial organization pattern among individuals (Figure S3C). Two‐dimensional plot and stress value were obtained using the “metaMDS” function in the “vegan” package (Oksanen et al., 2019), with Bray–Curtis dissimilarity as input and 25 random starting configurations. All three approaches yielded qualitatively similar results (Figure S3).

Following 2D ordination by PCA, clustering was performed on pairwise Euclidean distances of CHC composition between individuals to further characterize the spatial heterogeneity. Using the function “hopkins” in the “clustertend” package (Luo & Zeng, 2015), the clustering tendency was first examined by the Hopkins statistics (ranging from 0 to 0.5), with smaller values indicating the presence of spatial patchiness. The clustering tendency was cross‐validated by a visual approach (Figure S5A), implemented in the function “fviz_dist” from the “factoextra” package (Kassambara & Mundt, 2017). The optimal number of clusters was adopted using the majority rule, after comparing 30 results (Table S3) obtained by the function “fviz_nbclust” and “NbClust” in the package in the “factoextra” (Kassambara & Mundt, 2017) and “NbClust” package (Niknafs, Ghazzali, Boiteau, & Niknafs, 2015), respectively. Partition clustering by K‐means algorithm was performed on the CHC proportions, using the function “eclust” in the “factoextra” package (Kassambara & Mundt, 2017) with 50 random starting configurations. The performance of clustering was examined by silhouette width (Figure S5B), which captured both the within‐cluster compactness and between‐cluster separation. The silhouette indices ranged from −1 to 1, with increasing reliability of cluster assignment.

To identify the candidate CHCs that explained most of the between‐sample variation, variation retained by the principal components was partitioned using the “fviz_contrib” function in the “factoextra” package (Kassambara & Mundt, 2017). Briefly, the contribution of individual CHC to a given principal component was calculated as proportion of squared loading coefficients to the sum of squares. Using the function “fviz_pca_var” in the “factoextra” package (Kassambara & Mundt, 2017), variable map was constructed to visualize the correlation between candidate CHCs and (a) principal components, (b) discrete chemical clusters, (c) species, and (d) sex within a species. Metric‐based plots were produced using the “ggplot2” package (Wickham et al., 2018), and all statistical analyses were conducted in R studio (R Core Team, 2018).

## 3 RESULTS

### 3.1 The chemosensory system of *D. prolongata* shows a recent increase in sexual dimorphism

In most *Drosophila* species, including the closest relatives of *D. prolongata*, each foreleg carries ~30 chemosensory bristles in females and ~50 in males; the number and locations of these bristles are stereotypical and largely conserved across most of the genus. In contrast, the forelegs of *D. prolongata* males carry several hundred chemosensory bristles (Figure 1d), while the female forelegs have the same chaetotaxy as in the other related species of *Drosophila* (Figure 1b–d).

### 3.2 The CHC profile of *D. prolongata* shows increased sexual dimorphism

We used GC‐MS analysis to identify CHC compounds in male and female *D. fuyamai*, *D. rhopaloa*, *D. carrolli*, *D. kurseongensis*, and two strains of *D. prolongata*. Across all samples, we identified 27 distinct chromatogram peaks (Figure 2 and Figure S1) and determined the chemical identities of their corresponding compounds (Table 1). All these compounds have been identified previously in *D. melanogaster*. With the exception of the well‐known male‐specific cis‐vaccenyl acetate (cVA; Butterworth, 1969), we did not detect any qualitative sexual dimorphism, that is, the remaining 26 peaks were observed in both males and females (Table S1). These CHCs ranged from 21 to 29 carbons and fell into three chemical classes—linear alkanes, methyl‐branched alkanes, and mono‐unsaturated alkenes (monoenes). Monoenes with the same carbon number could be further divided into three positional isomers based on the location of the double bond (Table 1).

We quantified the relative abundance of each of 26 identified CHCs as a proportion of the total CHC blend of an individual. The compositional representation of CHCs was highly uneven for all species (Table S2). For example, among *D. prolongata* Bavi males, the most abundant CHC, 9‐pentacosene, contributed on average 46.6% to the total CHC blend (*SD* = 7.0%, median = 48.5%), while the 16 least abundant CHCs contributed <1% each. Consistent with other studies, the CHC blend was dominated by odd‐numbered CHCs, within which monoenes were the major constituents across all groups. Notably, for monoenes with the same carbon number, we discovered that the 9 isomers, especially 9‐tricosene and 9‐pentacosene (23 C and 25 C, respectively), were the major structural isomers in all species except in *D. fuyamai*.

**Figure 3:**
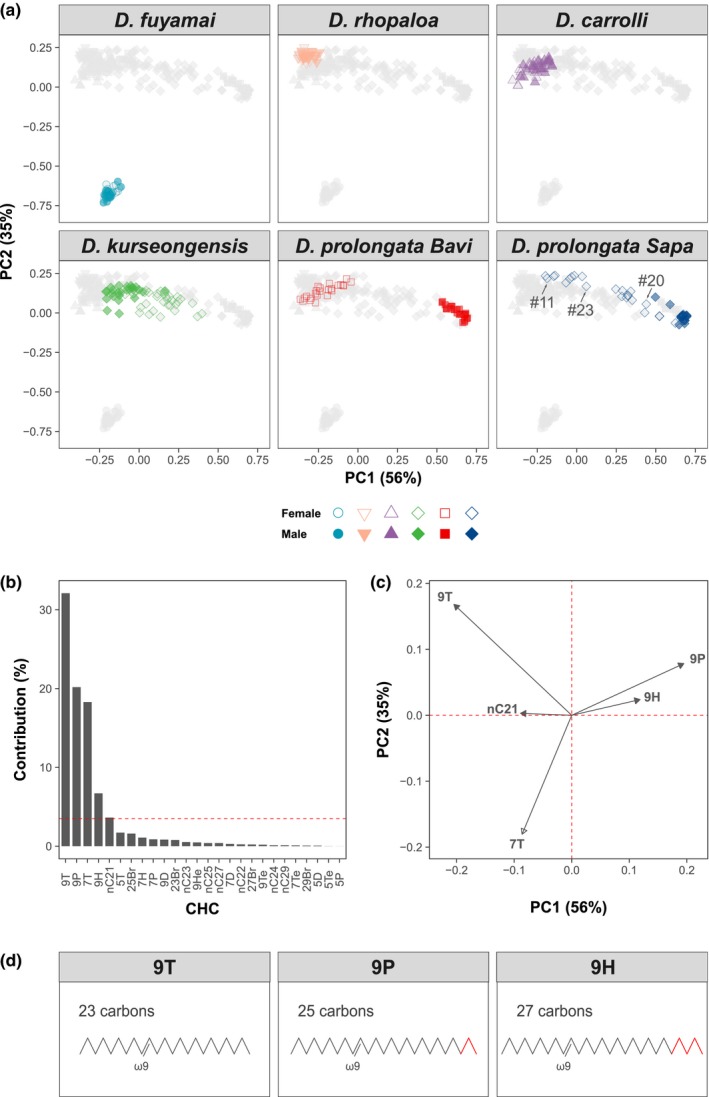
*Drosophila prolongata* males have a unique CHC profile. (a) Principal component analysis of CHC profiles of different species and sexes. Each species is shown in a different color and shape (see legend below). Each panel shows all individuals of all species, with the focal species highlighted in its assigned color and the remaining species shown in light gray. Females are represented by open symbols and males by filled symbols. The coordinates of individual points are loading scores after projecting all CHC components on the first two principal components. The variation explained by each PC is shown in parentheses. Arrows indicate female individuals ## 11, 23 and 20, respectively (also see Figure 4b). Note that the CHC profile of *D. prolongata* males distinguishes them both from conspecific females and from the males and females of other species. (b) Contributions of individual CHCs to the species‐ and sex‐specific variation retained by PC1 and PC2. CHCs were ordered by their association with PC1 and PC2. Dotted red line represents the null expectation that all CHCs contribute equally. (c) Vector map of individual CHCs on xy‐plane defined by PC1 and PC2. Only CHC candidates with above‐average contributions are displayed. For each CHC, the arrowhead indicates the direction of most rapid increase in its amount. The correlations among CHCs, or between CHCs and principal components (shown by red dotted lines), are represented by vector angles: positively correlated variables are indicated by acute angles, negatively correlated variables by obtuse angles, and uncorrelated by right angles. The relative contribution of each CHC to the variation explained by PC1 and PC2 is proportional to the length of the arrow. (d) Schematic illustration of the chemical structure of three related CHCs that contribute the most to sexual dimorphism in *D. prolongata*. 9P, 9T, and 9H share the same double bond at position 9 but differ in chain length (highlighted in red)

In principal component analysis, over 90% of between‐sample variation was captured by the first two PCs, with different species and sexes occupying different parts of the principal component space (Figure 3a). The clustering pattern was essentially unchanged if we used log‐contrast transformation prior to the multivariate analysis (Figure S3A), included cVA (Figure S3B), or used nonmetric multidimensional scaling (Figure S3C). Both sexes of *D. fuyamai*, the most distantly related species in our analysis, were found in the bottom‐left corner of the PCA plot, well separated from all other species. *D. rhopaloa* and *D. carrolli*, the two closest relatives of *D. prolongata*, clustered together in the top‐left corner, and also showed little or no sexual dimorphism. In contrast, the CHC profile of the *D. prolongata* Bavi strain was strongly sexually dimorphic: The females clustered together with both sexes of *D. rhopaloa* and *D. carrolli*, while the males formed their own well‐separated cluster in the top‐right corner (Figure 3a). *Drosophila kurseongensis* occupied the intermediate area between these two clusters and showed a moderate level of sexual dimorphism. The direction of sexual dimorphism in *D. kurseongensis* is reversed relative to *D. prolongata*, with females clustering to the right of males in the former and to the left of males in the latter.

### 3.3 Sexual dimorphism is variable in *D. prolongata*

In contrast to other species and sexes, the CHC profiles of *D. prolongata* Sapa females were highly variable, spanning the area from the top‐left to the top‐right cluster (Figure 3a). *Drosophila prolongata* Sapa males clustered together with the *D. prolongata* Bavi males. This pattern indicated that some Sapa females were similar to *D. prolongata* Bavi females, some others were similar to conspecific males, and many had CHC profiles that were intermediate between Bavi males and females. This polymorphism will be discussed below.

### 3.4 Dimorphic CHC profiles are caused by sex‐biased proportions of long and short monoenes

In *D. prolongata*, CHC composition is sexually dimorphic due to the unique CHC profile of males. To identify specific CHCs driving this difference, we partitioned the variance explained by the first 2 PCs. Five individual CHCs—n‐heneicosane (nC21), 9‐tricosene (9T), 7‐tricosene (7T), 9‐pentacosene (9P), and 9‐heptacosene (9H)—had higher than expected contributions to this variance (Figure 3b). To infer the relationship between the abundance of these CHCs and the clustering of individuals by species and sex on PCA plots (Figure 3a), we mapped the candidate CHCs to the coordinates defined by the first two PCs (Figure 3c). 7T was highly and negatively correlated with PC2, suggesting it was the major discriminator distinguishing *D. fuyamai* from the other species, where 9‐monoene isomers prevailed. Indeed, we found that 7T had much higher abundance in *D. fuyamai* than in the other species (Figure S4). Remarkably, PC1 was negatively correlated with short‐chain CHCs (9T, 7T, and nC21), but positively correlated with long‐chain CHCs (9P and 9H). This suggested a trade‐off between short and long CHCs along PC1. Specifically, overrepresentation of long‐chain CHCs and underrepresentation of short‐chain CHCs characterized the top‐right cluster, where males of *D. prolongata* resided. On the other hand, the top‐left cluster, which contains the females of *D. prolongata* and both sexes of *D. rhopaloa* and *D. carrolli*, is characterized by overrepresentation of short‐chain and underrepresentation of long‐chain CHCs.

**Figure 4:**
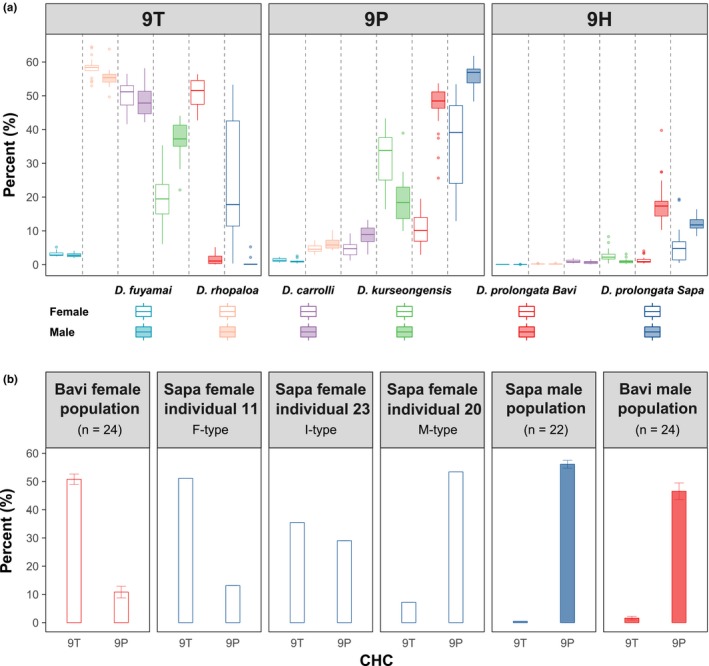
Sexual dimorphism and intraspecific variation in *Drosophila prolongata* are explained by the 9T/9P ratio. (a) Boxplots of the proportions of 9T, 9P, and 9H relative to total CHCs. Species are color‐coded and separated by dashed lines. Female individuals are in open bars and males in filled bars. (b) Bar plots of the proportions of 9T and 9P from two strains of *D. prolongata*. Both sexes of the Bavi strain and males of the Sapa strain are population samples, with sample size (*n*) indicated. Females ## 11, 20, and 23 from the Sapa strain are individual samples and are representative of the female‐type (F‐type), male‐type (M‐type), and intermediate‐type (I‐type)

To further assess whether sexual dimorphism within species and divergence between species could be attributed to specific short and long monoenes, we compared the relative proportions of the three candidates that constitute a homolog series (9T, 9P, and 9H), out of the total CHC blend, across all groups (Figure 4a). As expected, relative levels of these candidate CHCs were roughly equal between sexes in the three chemically monomorphic species: *D. fuyamai*, *D. rhopaloa*, and *D. carrolli*. In chemically dimorphic species, however, the relative proportions of 9T, 9P, and 9H were clearly different between sexes. In *D. prolongata*, especially in the Bavi strain, the longer‐chain 9P was much more abundant in males than in females, while the shorter‐chain 9T showed the opposite pattern (Figure 4a). This dimorphism was reversed in *D. kurseongensis*, where males had a higher proportion of 9T and females a higher proportion of 9P.

### 3.5 Intraspecific polymorphism in *D. prolongata* is also attributable to the long:short monoene ratio

Females of the *D. prolongata* Sapa strain show unusually high variation in the relative proportions of 9T and 9P (Figure 4a), reminiscent of their intermediate CHC phenotypes along PC1 revealed by PCA (Figure 3a). By analyzing individual females, we found that the variable ratio between 9T and 9P accounts for this sex‐limited polymorphism. For example, Sapa female #11, which resided in the top‐left cluster (Figure 3a), resembles females of the *D. prolongata* Bavi strain in having a high 9T:9P ratio (Figure 4b). In contrast, Sapa female #20, which resided in the top‐right (“male”) cluster in PCA, resembles conspecific males of both strains in having a low 9T:9P ratio (Figure 4b). Finally, Sapa female #23 was found in the intermediate region between the two PCA clusters and had roughly equal proportions of 9T and 9P. We categorized *D. prolongata* Sapa females into female‐like (F‐type), intermediate (I‐type), and male‐like (M‐type) CHC classes based on their 9T:9P ratio (Figure 4b).

Intriguingly, 9T and 9P have similar chemical structure, with the same double‐bond position; their only difference is the length of the carbon backbone (Figure 3d). This structural similarity points to a single biosynthetic pathway that could explain both the sexual dimorphism and the intraspecific variation in *D. prolongata*, as we discuss below.

## 4 DISCUSSION

Sexual selection theories have extensively discussed how interactions between signals and preferences can drive the evolution of sexual dimorphism (Andersson, 1994). These theories can best be tested in models that show recent and simultaneous emergence of sexual dimorphism in signal production and perception. In this respect, *D. prolongata* has the potential to be an excellent model. Its exaggerated sexual dimorphism in foreleg morphology is clearly of very recent origin and contributes directly to courtship behavior and mating success (Setoguchi, Kudo, Takanashi, Ishikawa, & Matsuo, 2015; Setoguchi et al., 2014). This evolutionary change is accompanied by a greatly increased number of chemosensory bristles exclusively in males and, as we show here, by an equally recent change in the male‐specific CHC profile, suggesting potential coevolution between pheromone production and perception.

Reconstructing the origin of sexual dimorphism requires a mechanistic understanding of the pathways that produce the dimorphic traits. The biosynthetic pathway responsible for CHC synthesis in *Drosophila* is the ultimate source of all sex‐specific and sexually monomorphic compounds (Howard & Blomquist, 2005). In most populations of *D. melanogaster*, sexually dimorphic expression of elongase and desaturase enzymes (*eloF* and *desatF*) leads to sex‐biased abundance of several CHCs including the female‐enriched 7,11‐HD and the male‐enriched 7‐Tricosene (7T; Antony & Jallon, 1982; Chertemps et al., 2007; Chertemps et al., 2006). Interestingly, we find that 7T is enriched in *D. fuyamai*, compared with other species, but this enrichment is sexually monomorphic (Figure 3a,c and Figure S4). Female‐enriched alkadienes are also found in most relatives of *D. melanogaster* (Jallon & David, 1987), which show female‐biased expression of *desatF* (Shirangi et al., 2009) and *eloF* (Combs et al., 2018). This sexual dimorphism has an important functional role, for example, evolutionary changes in *eloF* expression contribute to strong behavioral isolation between the sibling species *D. simulans* and *D. sechellia* (Combs et al., 2018). However, sexually dimorphic alkadiene production in *D. melanogaster* and its relatives appears to have evolved at the base of the *melanogaster* species subgroup, with most species differences caused by secondary losses (Shirangi et al., 2009). In contrast, the male‐biased expression of long‐chain monoenes 9P and 9H in *D. prolongata*, at the expense of the shorter female‐biased 9T, has evolved more recently and provides a good alternative model for understanding the emergence of sexually dimorphic communication.

Based on the structure of the CHC synthesis pathway, we hypothesize that both the male‐biased abundance of 9P and 9H and the female‐biased abundance of 9T in *D. prolongata* could in principle be attributable to a simple genetic change. Namely, we hypothesize that male‐biased expression of an elongase enzyme that catalyzes the conversion of 9‐C24:1‐CoA into 9‐C26:1‐CoA and 9‐C28:1‐CoA could explain both the higher abundance of 9P and 9H and the lower abundance of 9T in males. Conversely, lower expression of this enzyme in females would lead to higher abundance of 9T and lower abundance of 9P and 9H. Under this model, the key evolutionary change would be a transition from sexually monomorphic to male‐biased carbon chain elongation in *D. prolongata* following its divergence from *D. carrolli* and *D. rhopaloa*. This change could be due to the gain of a new elongase gene, changes in the chemical activity of an existing enzyme, or, most simply, to a regulatory mutation that leads to male‐biased expression of an elongase that was expressed monomorphically in the ancestral condition. The female‐limited polymorphism in the relative proportions of 9P and 9T might also be explained by the same mechanism.

In summary, *D. prolongata* shows clear evidence of recently evolved sexual dimorphism in pheromone synthesis and an intriguing case of female‐limited polymorphism. Both the interspecific divergence and the intraspecific variation in CHC profiles involve a homolog series of three monoenes, 9T, 9P, and 9H, which likely share a common biosynthetic origin. The relatively close relationship between *D. prolongata* and the model species *D. melanogaster*, where a wide range of genetic tools is available, makes it an attractive model for investigating how sexual selection acts on the genome to generate new sex‐specific traits, and how signal production can co‐evolve with signal perception.

## CONFLICT OF INTEREST

None declared.

## AUTHOR CONTRIBUTIONS

Y.L. and A.K. conceived and designed the study. Y.L. collected the data. Y.L., Y.Z., and J‐P.F. analyzed data. J‐F.F and S.R supervised the project. Y.L. wrote the first draft of the manuscript. All authors reviewed and revised the manuscript.

## Data Availability

All raw data will be accessible on Dryad with the accession https://doi.org/10.25338/B86K7V.
