# Supplementary figures and images for: Evolution of sexually dimorphic pheromone profiles coincides with increased number of male‐specific chemosensory organs in Drosophila prolongata
Source: Ecol Evol. 2019 Nov 17;9(23):13608–18. doi: 10.1002/ece3.5819 (PMC6912897; doi:10.1002/ece3.5819)

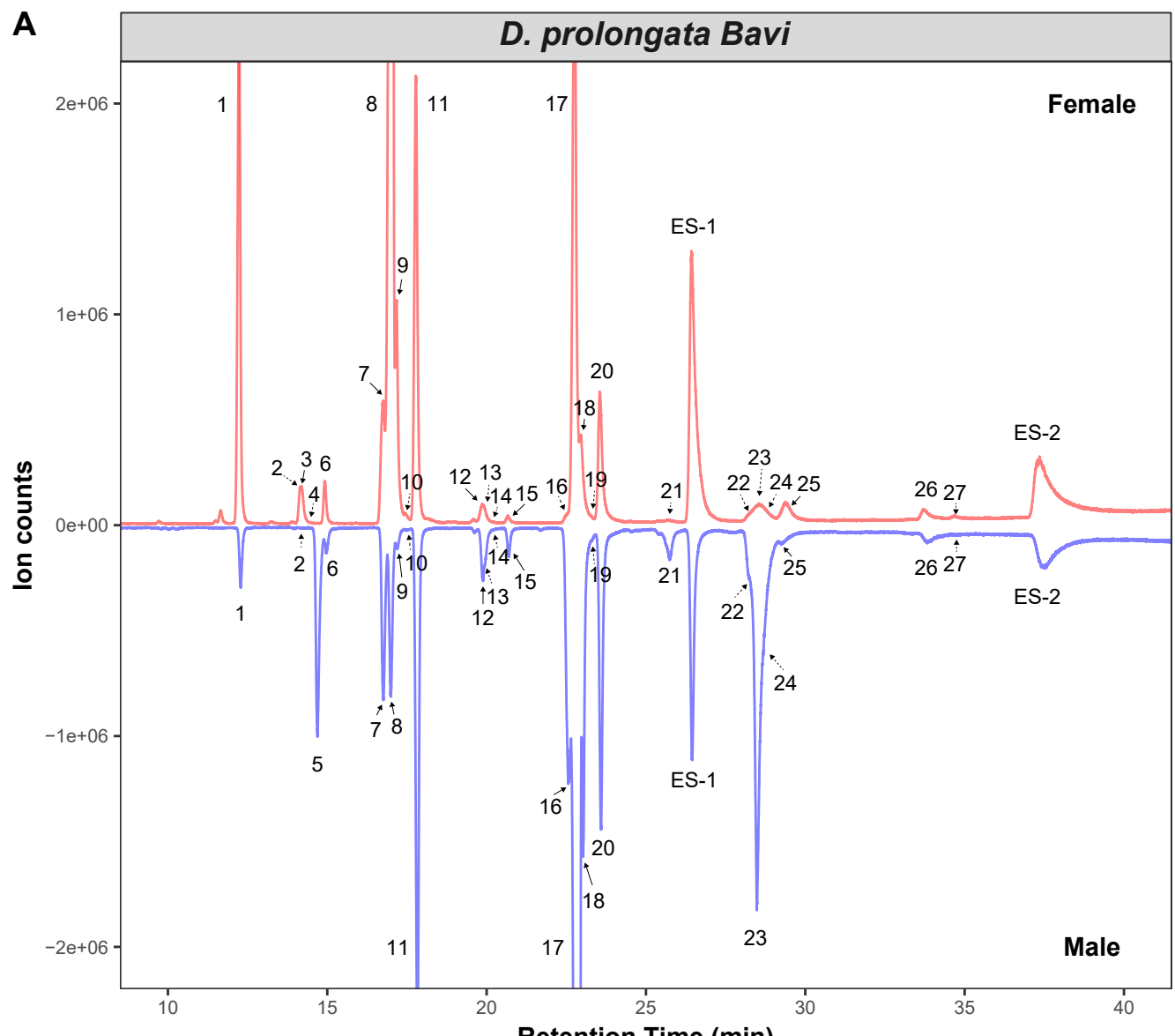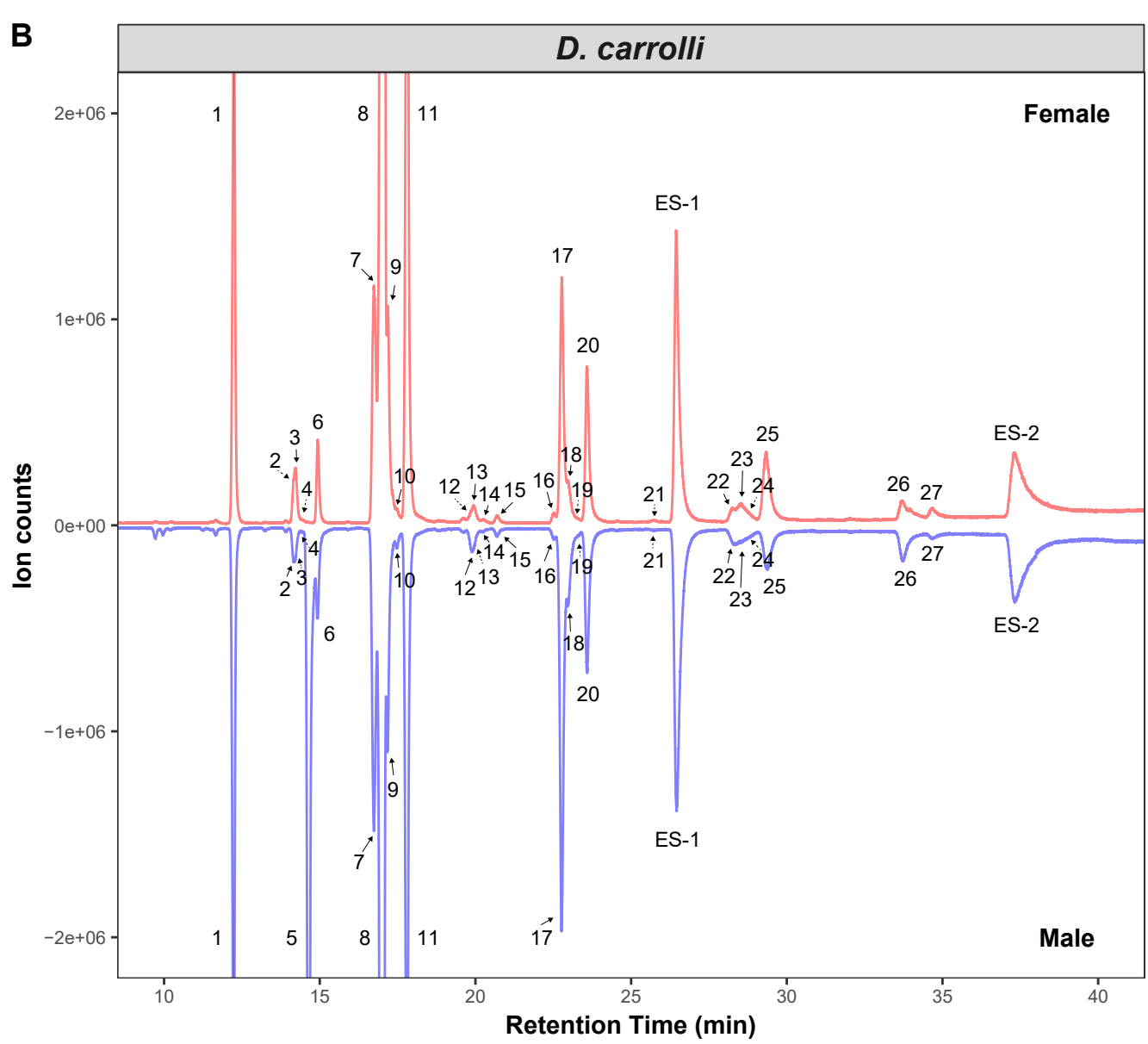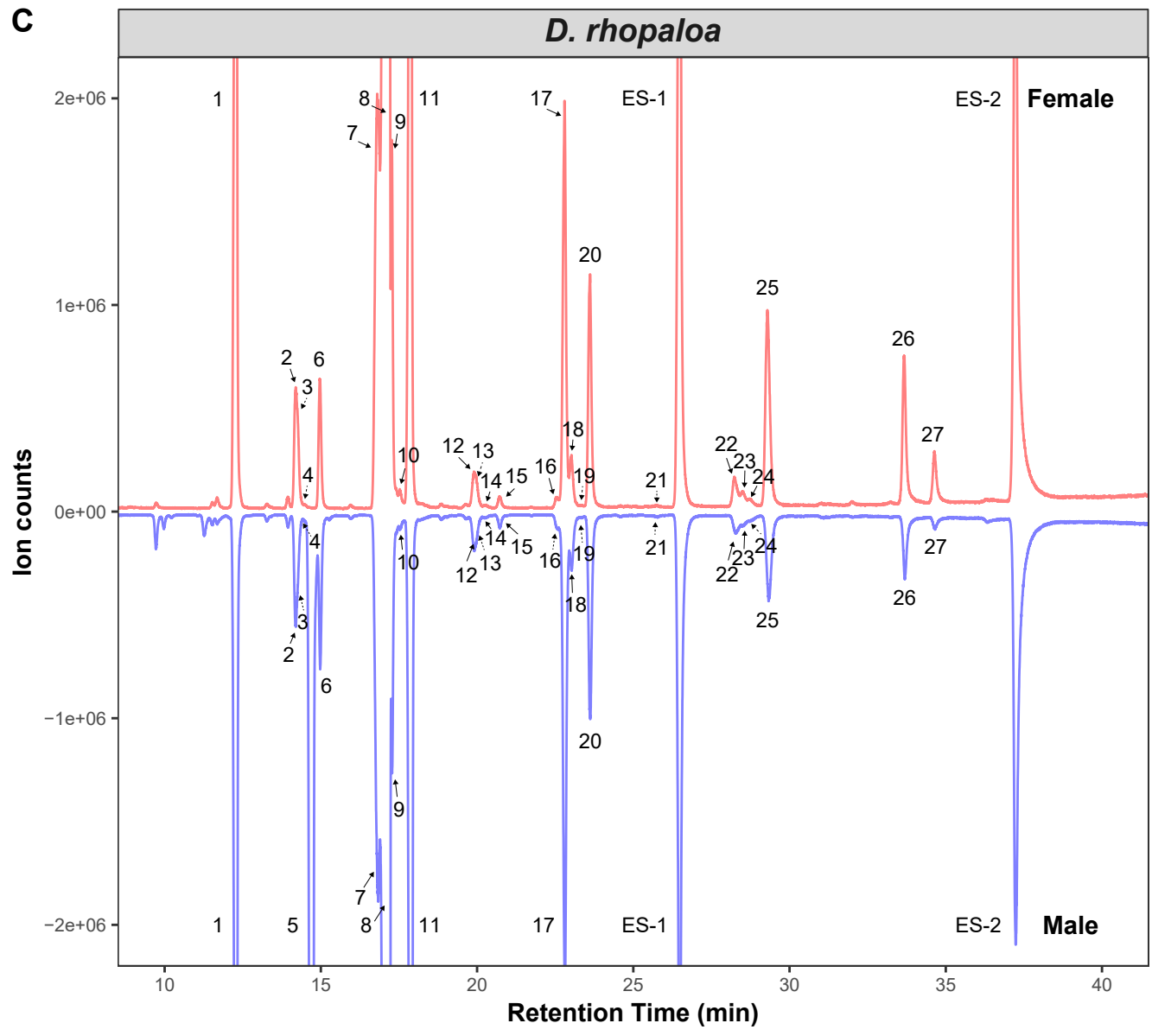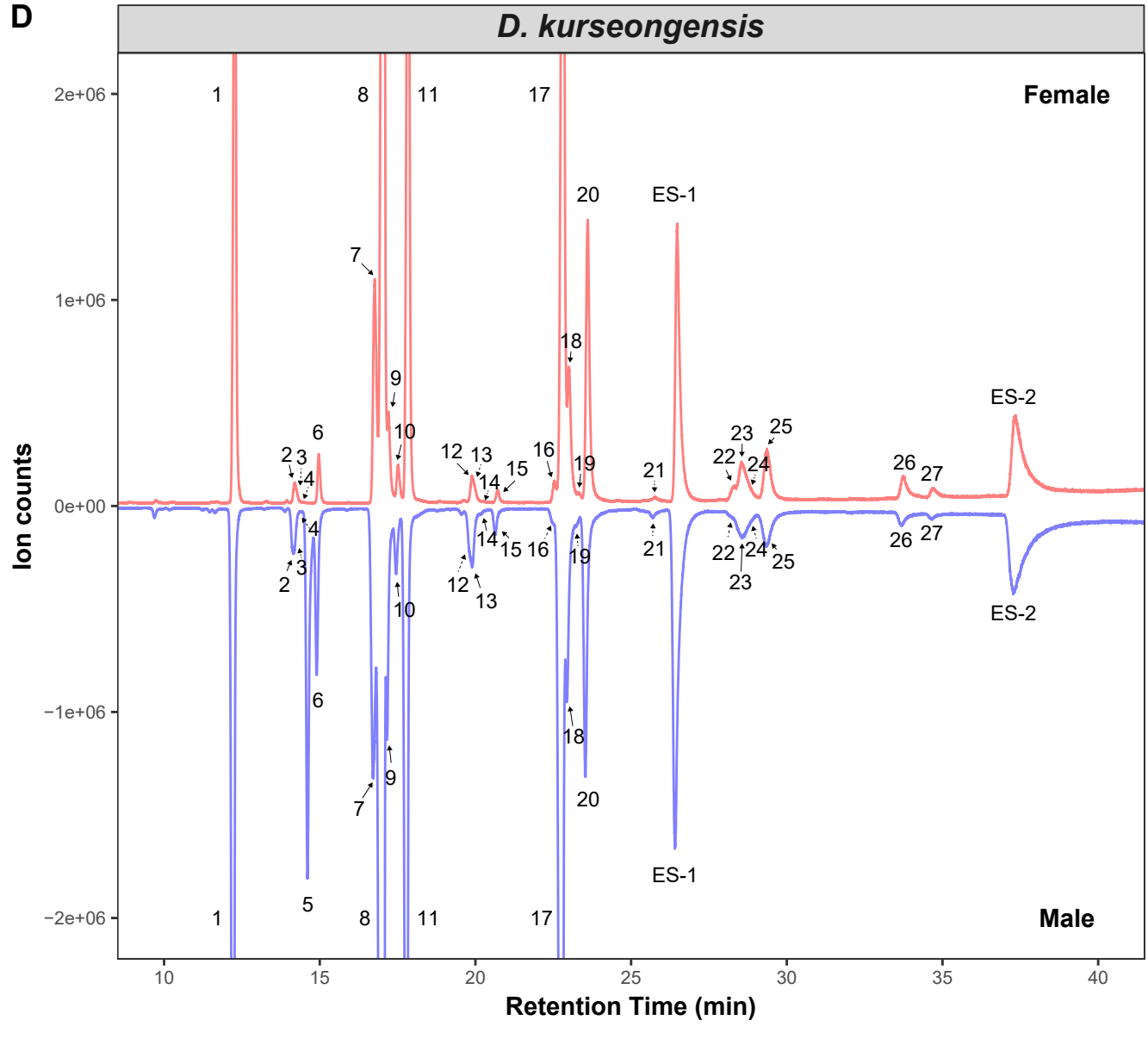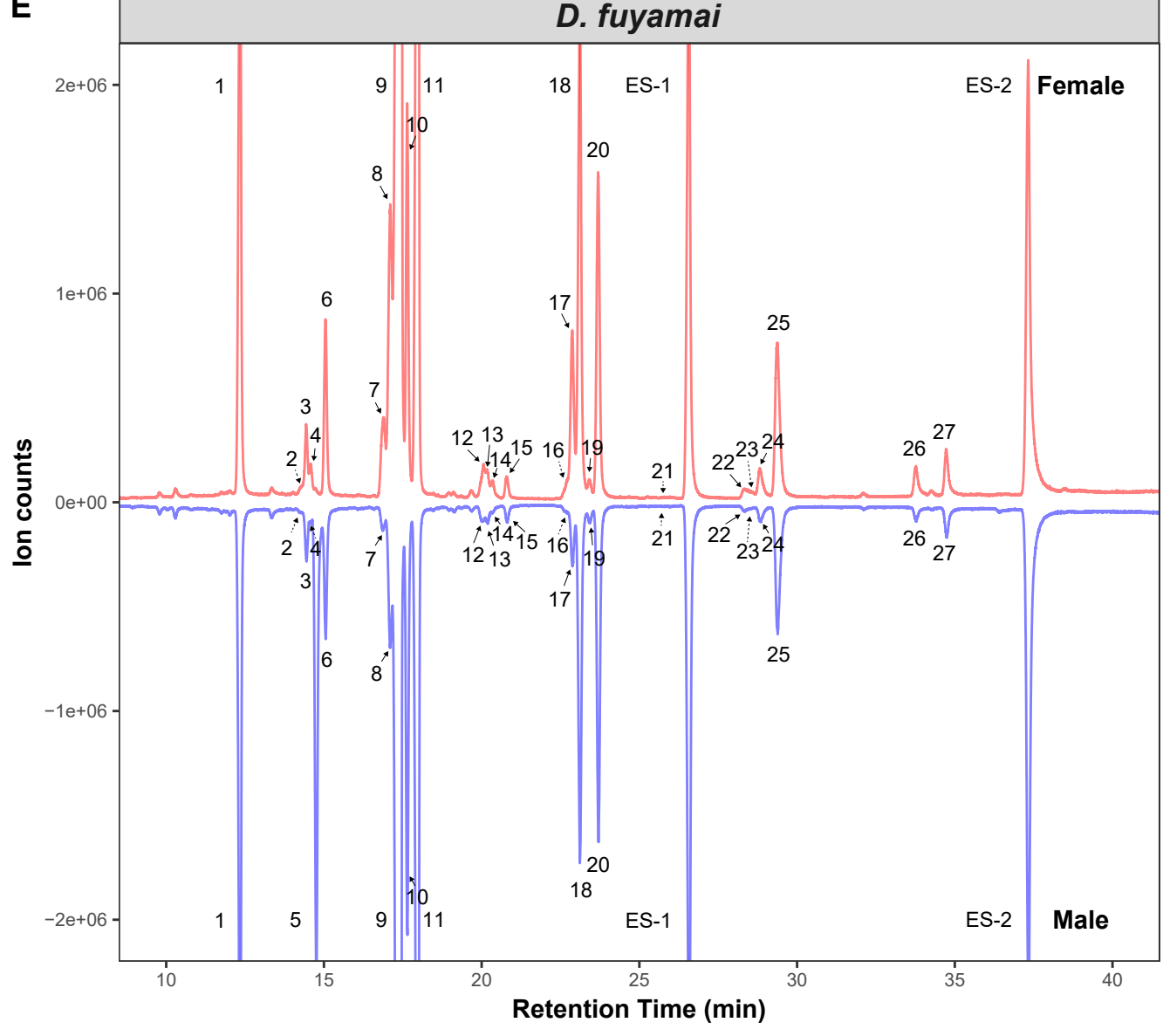

Supplement: Supplementary file 1 [file ECE3-9-13608-s001.pdf]

**A**

Female ○ ▽ △ ◇ □ ◇  
 Male ● ▽ △ ◇ □ ◇

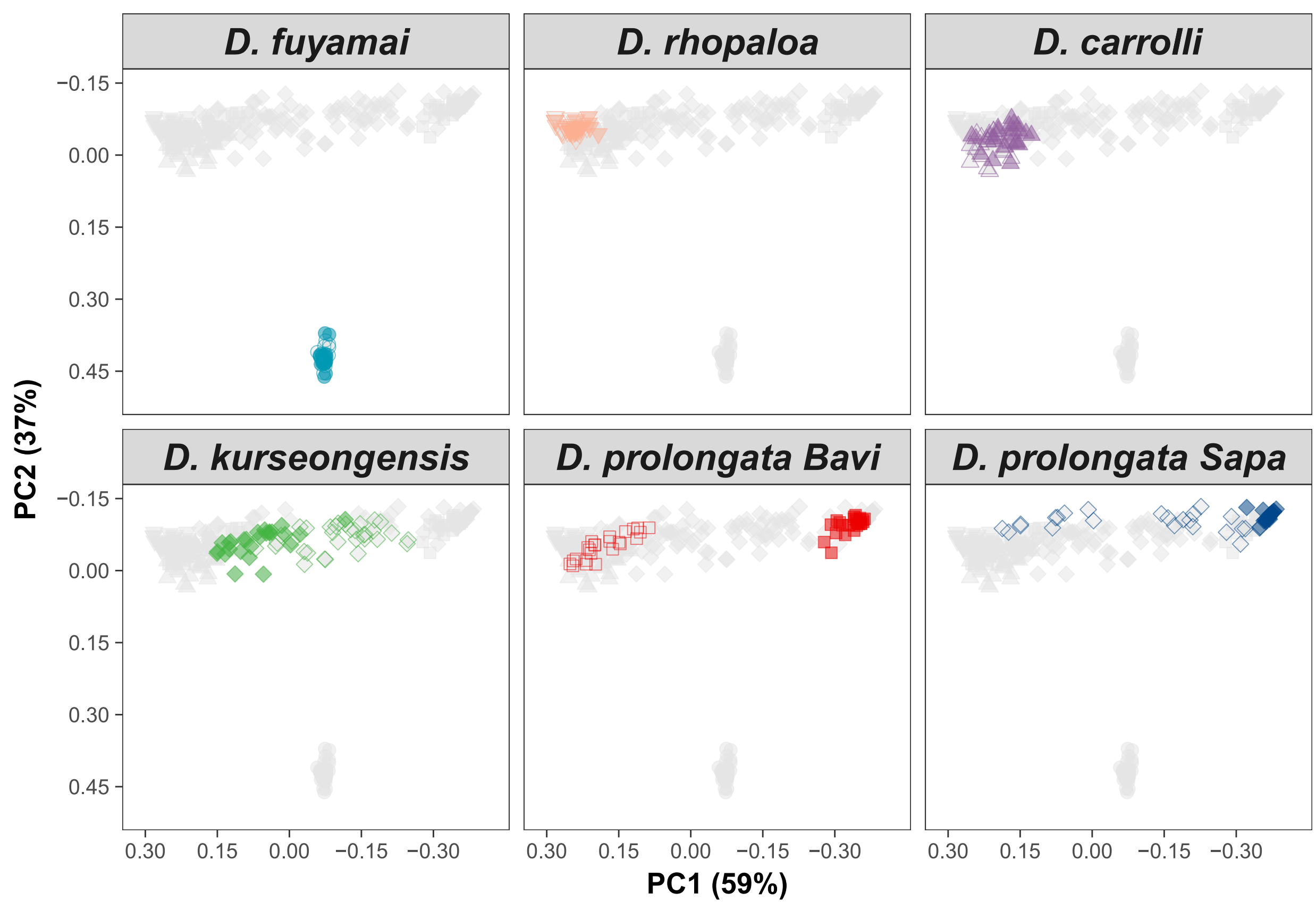**B**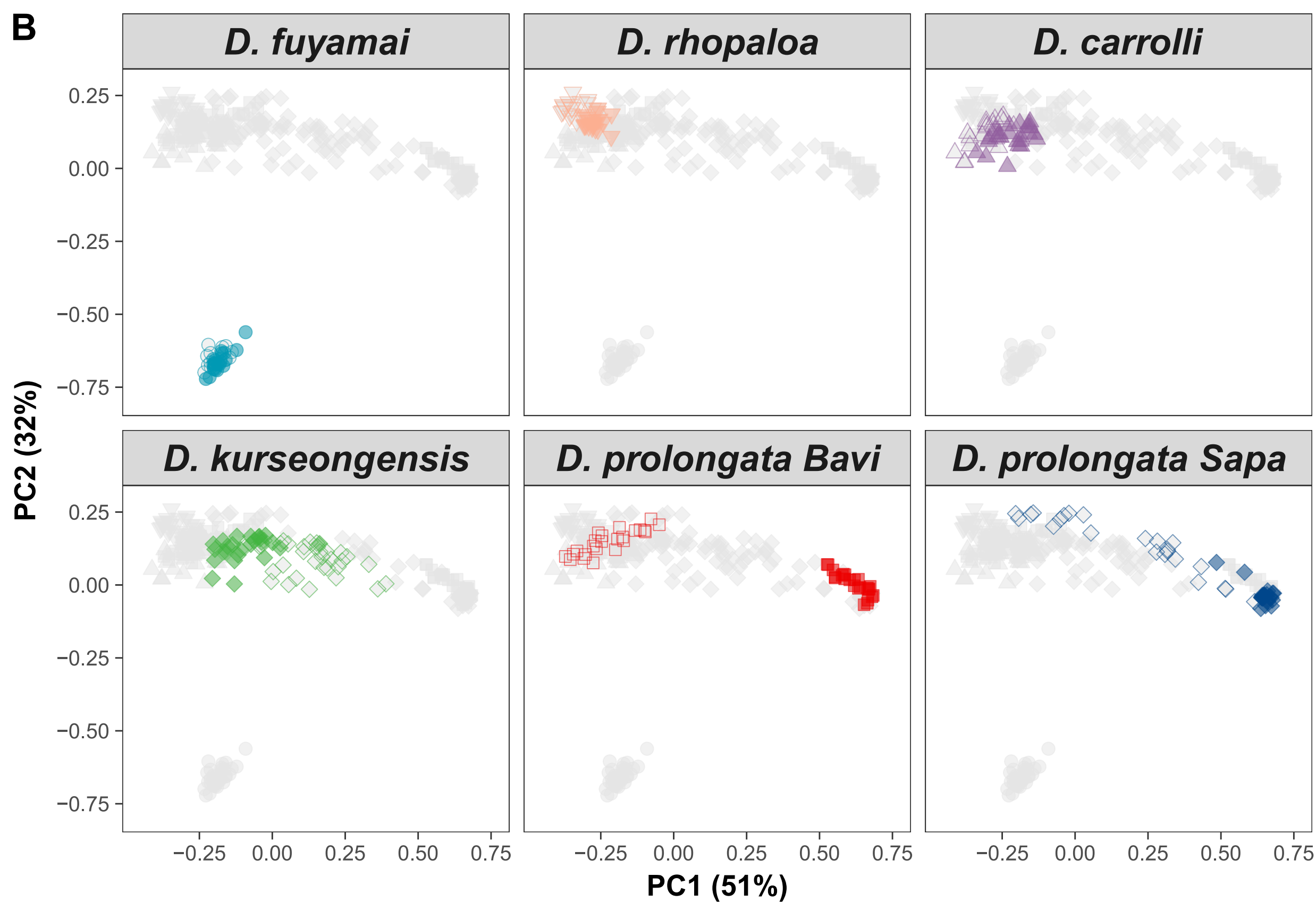**C**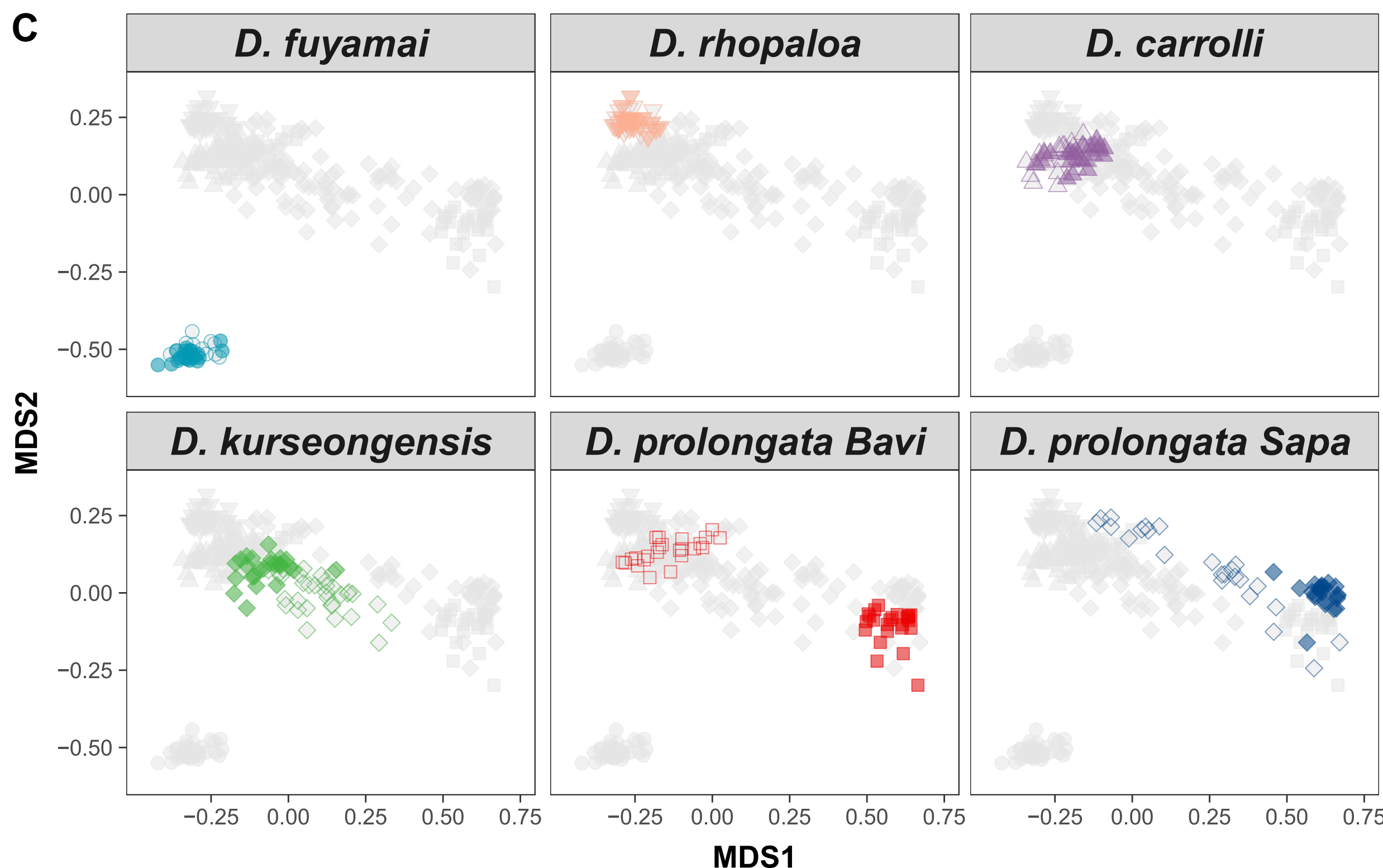

Supplement: Supplementary file 3 [file ECE3-9-13608-s003.pdf]

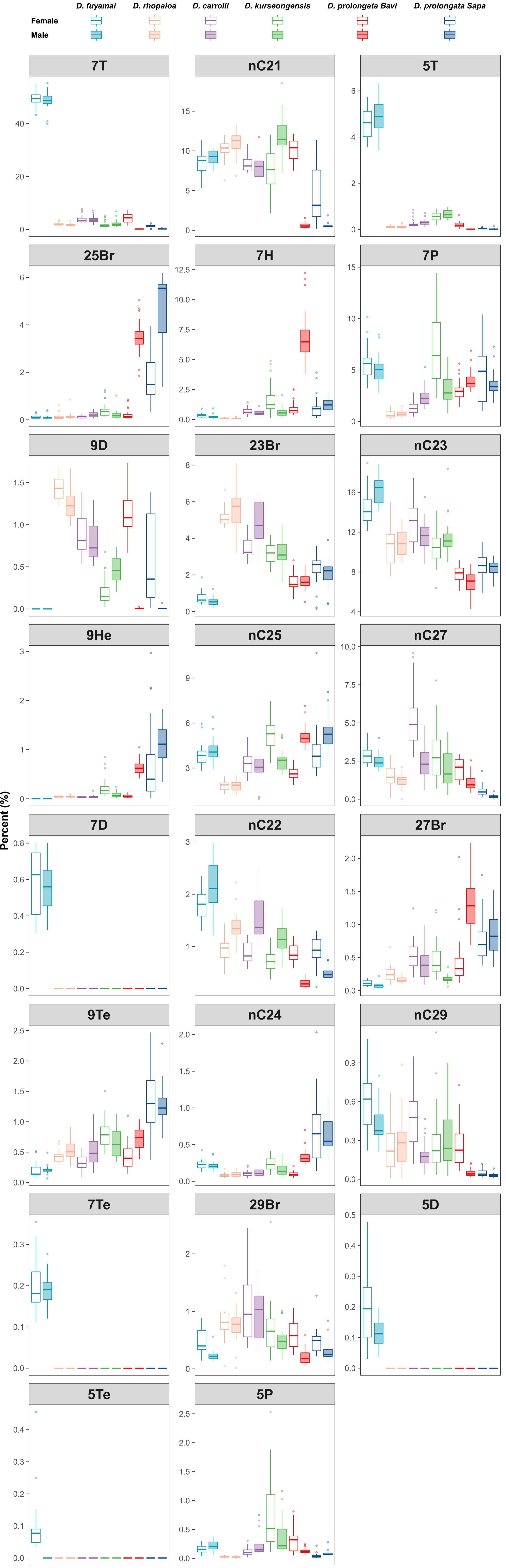

Supplement: Supplementary file 4 [file ECE3-9-13608-s004.pdf]

**A**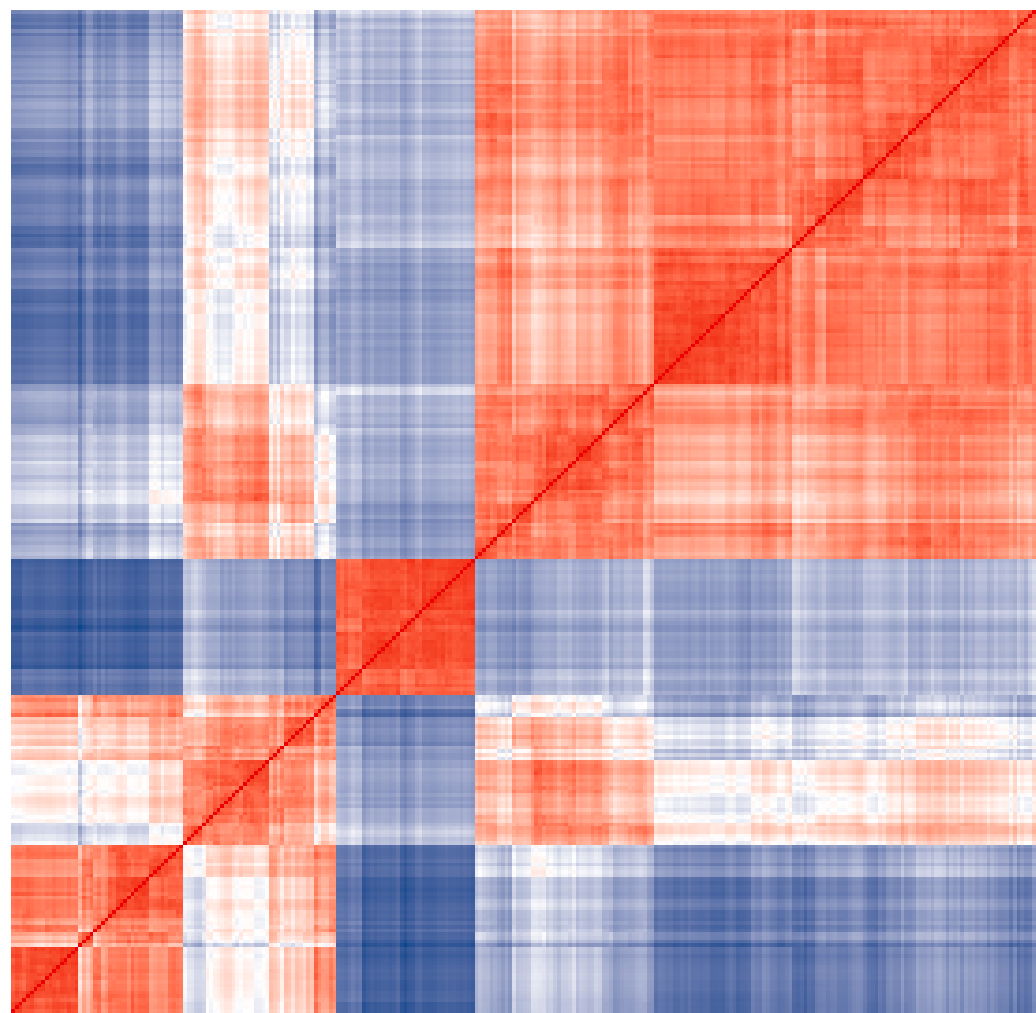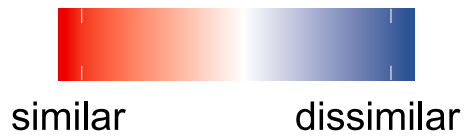**B**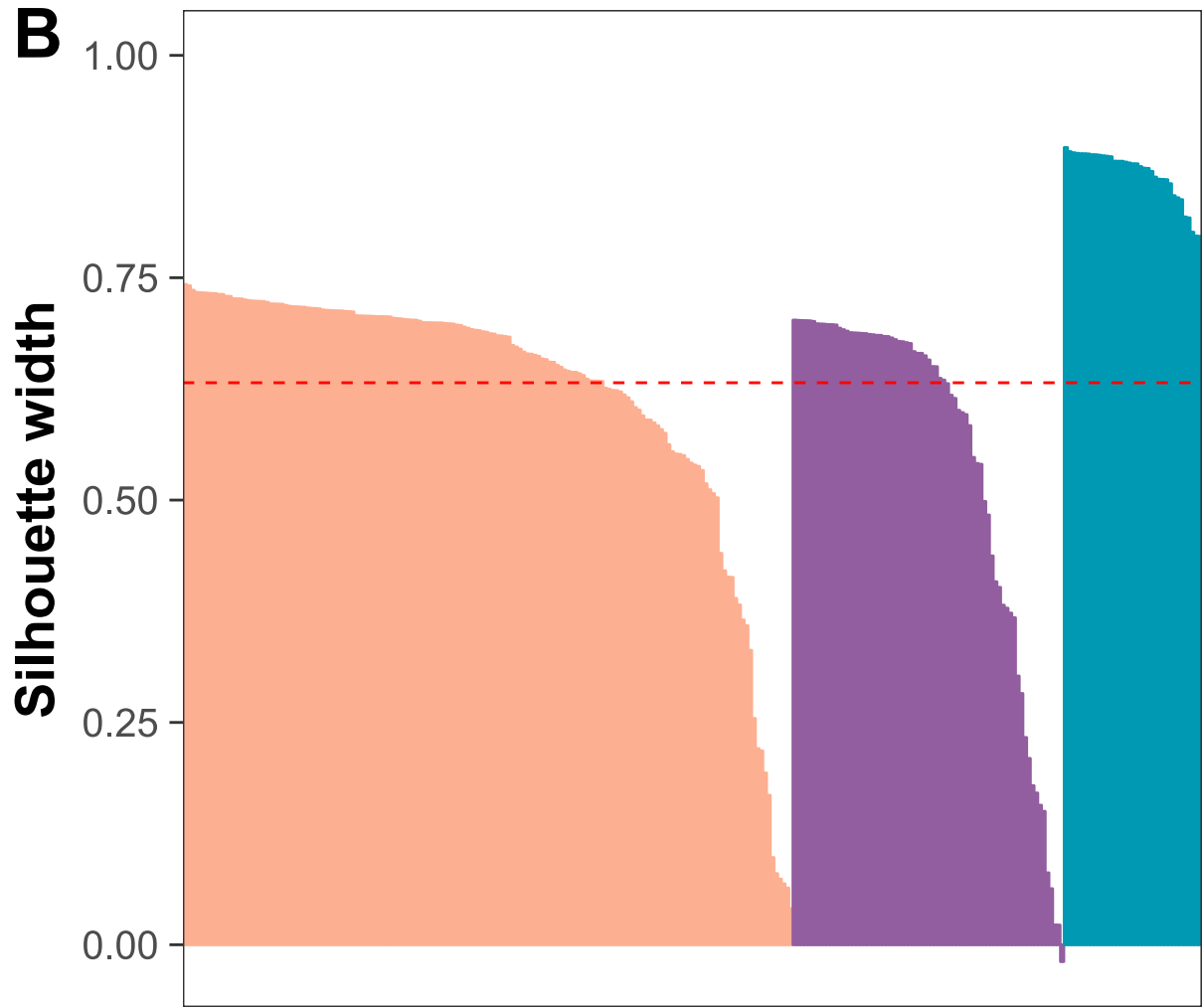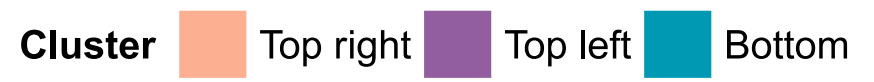

Supplement: Supplementary file 5 [file ECE3-9-13608-s005.pdf]
