# Supplementary material for: Evolution of sexually dimorphic pheromone profiles coincides with increased number of male‐specific chemosensory organs in Drosophila prolongata
Source: Ecol Evol. 2019 Nov 17;9(23):13608–18. doi: 10.1002/ece3.5819 (PMC6912897; doi:10.1002/ece3.5819)
Supplement: Supplementary file 6 [file ECE3-9-13608-s006.docx]

**SUPPLEMENTARY MATERIALS**

**Table S1.** **Qualitative** **expression^[[1]](#footnote-1)^ of CHCs in each species and sex**.

| **Compound** | ***D. prolongata Sapa*** | | ***D. prolongata Bavi*** | | ***D. carrolli*** | | ***D. rhopaloa*** | | ***D. kurseongensis*** | | ***D. fuyamai*** | |
| --- | --- | --- | --- | --- | --- | --- | --- | --- | --- | --- | --- | --- |
|  | **♀** | **♂** | **♀** | **♂** | **♀** | **♂** | **♀** | **♂** | **♀** | **♂** | **♀** | **♂** |
| **nC21** | + | + | + | + | + | + | + | + | + | + | + | + |
| **9D** | + | + | + | + | + | + | + | + | + | + | tr | tr |
| **7D** | tr |  | tr |  | tr | tr | tr | tr | tr | tr | + | + |
| **5D** | tr |  | tr |  | tr | tr | tr | tr | tr | tr | + | + |
| **cVA** |  | + |  | + |  | + |  | + |  | + |  | + |
| **nC22** | + | + | + | + | + | + | + | + | + | + | + | + |
| **23Br** | + | + | + | + | + | + | + | + | + | + | + | + |
| **9T** | + | + | + | + | + | + | + | + | + | + | + | + |
| **7T** | + | + | + | + | + | + | + | + | + | + | + | + |
| **5T** | + | + | + | + | + | + | + | + | + | + | + | + |
| **nC23** | + | + | + | + | + | + | + | + | + | + | + | + |
| **9Te** | + | + | + | + | + | + | + | + | + | + | + | + |
| **7Te** | tr | tr | tr | tr | tr | tr | tr | tr | tr | tr | + | + |
| **5Te** | tr | tr | tr | tr | tr | tr | tr | tr | tr | tr | + | tr |
| **nC24** | + | + | + | + | + | + | + | + | + | + | + | + |
| **25Br** | + | + | + | + | + | + | + | + | + | + | + | + |
| **9P** | + | + | + | + | + | + | + | + | + | + | + | + |
| **7P** | + | + | + | + | + | + | + | + | + | + | + | + |
| **5P** | + | + | + | + | + | + | + | + | + | + | + | + |
| **nC25** | + | + | + | + | + | + | + | + | + | + | + | + |
| **9He** | + | + | + | + | + | + | + | + | + | + | + | + |
| **27Br** | + | + | + | + | + | + | + | + | + | + | + | + |
| **9H** | + | + | + | + | + | + | + | + | + | + | + | + |
| **7H** | + | + | + | + | + | + | + | + | + | + | + | + |
| **nC27** | + | + | + | + | + | + | + | + | + | + | + | + |
| **29Br** | + | + | + | + | + | + | + | + | + | + | + | + |
| **nC29** | + | + | + | + | + | + | + | + | + | + | + | + |

**Table S2.** **Quantitative** **expression^[[2]](#footnote-2)^ of CHCs in each species and sex**.

| **Compound** | **Amount (ng/fly, mean ± SEM)** | | | | | | | | | | | |
| --- | --- | --- | --- | --- | --- | --- | --- | --- | --- | --- | --- | --- |
|  | ***D. prolongata Sapa*** | | ***D. prolongata Bavi*** | | ***D. carrolli*** | | ***D. rhopaloa*** | | ***D. kurseongensis*** | | ***D. fuyamai*** | |
|  | **Female** | **Male** | **Female** | **Male** | **Female** | **Male** | **Female** | **Male** | **Female** | **Male** | **Female** | **Male** |
| **nC21** | 108.7±22.5 | 17.7±2.7 | 336.9±30.7 | 23.7±2.9 | 205.3±12.5 | 185.8±15.6 | 191.7±12 | 198.6±13.1 | 210±17.5 | 298.2±22 | 129.9±11.2 | 122.1±8.7 |
| **9He** | 14.2±3.1 | 0.2±0.1 | 38.5±4.3 | 0.3±0.0 | 21.7±1.5 | 18.6±1.8 | 27.6±1.8 | 23.4±1.9 | 6±1 | 11.8±1.1 | tr | tr |
| **7He** | tr | 0 | tr | 0 | tr | tr | tr | tr | tr | tr | 9.3±1.1 | 7.6±0.8 |
| **5He** | tr | 0 | tr | 0 | tr | tr | tr | tr | tr | tr | 3.2±0.6 | 1.5±0.2 |
| **cVA** | 0 | 244.9±20.3 | 0 | 84±7.3 | 0 | 220±18.9 | 0 | 288.7±20.4 | 0 | 66.6±10 | 0 | 110.7±21.8 |
| **nC22** | 18.9±2.2 | 14.9±1.2 | 30±3.3 | 11.3±0.8 | 21.4±1.5 | 34.7±2.3 | 17.7±1 | 24.8±1.3 | 20±1.8 | 27.9±1.8 | 27.7±2.6 | 28.9±2.4 |
| **23Br** | 46.7±5.1 | 64.5±7.1 | 58.3±8.1 | 63.9±4.9 | 85.1±5.3 | 116.5±12.3 | 98.2±5.5 | 100.1±5.6 | 86.6±5.9 | 83.8±6.8 | 11.7±1.6 | 7.7±0.9 |
| **9T** | 593.2±116.2 | 15.1±8 | 1684.5±147.6 | 62±12.6 | 1221.6±62.6 | 1168.1±102.9 | 1119.6±70.5 | 1011.1±58.7 | 561.3±47.8 | 936.8±70.3 | 48.1±3.9 | 37.8±2.7 |
| **7T** | 26.4±2.6 | 6.6±1 | 133.9±12 | 7±0.7 | 95.3±9 | 89.2±9.8 | 37.6±2 | 33.7±2.6 | 47.7±5.8 | 54.4±5.1 | 745.4±57 | 641.7±39.1 |
| **5T** | 0.7±0.1 | 0.6±0.2 | 5.9±0.7 | 0.6±0.1 | 6.7±1 | 7±0.8 | 2.2±0.2 | 2±0.3 | 15.5±1.5 | 16.8±1.6 | 70.6±6.3 | 64.5±4.3 |
| **nC23** | 169.3±14.3 | 259.4±21 | 256.8±21.8 | 267.1±16.8 | 314.8±20.1 | 274.7±24.3 | 197.6±11.3 | 198.4±9.7 | 282±15.6 | 284±15.6 | 214.5±13.9 | 216.7±15.9 |
| **9He** | 24.7±2.3 | 40.5±4.1 | 15.7±2.8 | 29.1±2.5 | 8±0.8 | 13.1±1.8 | 8.2±0.6 | 9.9±1 | 22.9±2.3 | 17.3±1.8 | 3.3±0.7 | 2.9±0.3 |
| **7He** | tr | tr | tr | tr | tr | tr | tr | tr | tr | tr | 3.1±0.4 | 2.6±0.3 |
| **5He** | tr | tr | tr | tr | tr | tr | tr | tr | tr | tr | 1.8±0.6 | tr |
| **nC24** | 10.8±1 | 19.4±2 | 3.3±0.4 | 13±1 | 2.6±0.2 | 2.7±0.3 | 1.6±0.1 | 1.7±0.2 | 6.6±0.6 | 3.9±0.4 | 3.6±0.5 | 3±0.4 |
| **25Br** | 31.8±3.1 | 145.1±13.2 | 9.3±2.6 | 134.9±10.3 | 2.9±0.3 | 5.2±0.7 | 2.6±0.5 | 3.1±0.8 | 11.1±1.5 | 5.9±1.2 | 2.1±0.4 | 1.8±0.5 |
| **9P** | 624.2±47.5 | 1742.5±147 | 391.4±69.4 | 1798±118.2 | 111.1±12.3 | 216.8±25.9 | 92±7.9 | 113.3±9.7 | 866.6±63.2 | 500.2±52.4 | 21.7±2.8 | 14.4±2.5 |
| **7T** | 72.8±7.8 | 108.1±9.4 | 101.4±13.1 | 148.3±7.5 | 32.4±4.2 | 61.5±9.2 | 12.7±1.5 | 13.4±1.6 | 175.9±16.9 | 81.8±9.4 | 89.4±10.6 | 70.3±9.4 |
| **5T** | 0.8±0.2 | 2.7±0.4 | 9.5±1.4 | 4.7±0.3 | 2.9±0.4 | 6±1.4 | 0.6±0.1 | 0.4±0.0 | 19.2±2.8 | 9.5±1.5 | 2.6±0.4 | 3±0.5 |
| **nC25** | 70.9±3.7 | 167.1±14.1 | 87.6±8.4 | 197±12.5 | 81.3±6.5 | 73±8.3 | 34.1±2.1 | 32.1±2.1 | 136.1±6.6 | 86.8±5.5 | 58.6±4.4 | 57.8±6.7 |
| **9He** | 10.3±2.1 | 35.2±3.7 | 2±0.4 | 25.7±2.4 | 0.7±0.1 | 0.9±0.1 | 0.9±0.1 | 0.8±0.1 | 6.5±1.2 | 2.2±0.4 | tr | tr |
| **27Br** | 13.5±1.2 | 27.9±3.5 | 18.5±5.3 | 53.7±5.6 | 12.8±1.3 | 9.9±1.4 | 5.1±0.7 | 3±0.3 | 12.1±1.6 | 4.4±0.4 | 1.7±0.2 | 1.1±0.3 |
| **9H** | 79.4±11.6 | 381.5±37 | 53.8±13.4 | 746.4±85.3 | 24.4±2.9 | 16.7±2.3 | 4.4±0.5 | 3.9±0.5 | 73.4±10 | 26.5±4.6 | 0.7±0.1 | 0.5±0.1 |
| **7H** | 15.8±2.2 | 36.5±2.8 | 36.2±8 | 275.3±28.4 | 16.6±2.3 | 13.6±2 | 2.1±0.2 | 1.9±0.3 | 45.7±6.5 | 16.9±2.9 | 5.5±1 | 3.8±1.1 |
| **nC27** | 11±1.7 | 6.1±1.1 | 70.8±10.4 | 44.2±6.7 | 129.1±13.5 | 54±6 | 29.8±3.7 | 22±2.5 | 77.1±8.4 | 48.7±6.9 | 44.2±4.2 | 34.6±4.4 |
| **29Br** | 8.3±0.6 | 10.4±2.1 | 20.2±2.6 | 8.4±1.2 | 24.8±3.6 | 21.5±2.3 | 16.7±2 | 13.4±1.3 | 17.3±1.7 | 12.4±1.2 | 7.4±1 | 3.4±0.6 |
| **nC29** | 1±0.1 | 0.9±0.1 | 8.2±1.2 | 1.9±0.2 | 11±1.3 | 4.3±0.6 | 5±1 | 5.4±1 | 7.1±0.8 | 7.5±1 | 10±1.3 | 6±0.9 |
| **Σ Linear** | 390.5±40.8 | 485.6±39.6 | 793.6±72.5 | 558.3±33.9 | 731.2±54.9 | 561±50.2 | 477.6±27.7 | 483.1±25.8 | 738.8±42.1 | 756.8±45 | 488.5±34.5 | 477±38.1 |
| **Σ Branched** | 100.3±7.9 | 247.8±22.8 | 106.3±16.7 | 260.9±19 | 123±8.8 | 126.9±9.7 | 122.6±7.3 | 119.6±6.7 | 127.2±8.7 | 106.5±8.3 | 22.9±2.6 | 14.5±2.1 |
| **Σ 9-Monoene** | 1345.9±121 | 2215±191.3 | 2186±220.5 | 2661.5±181.3 | 1360.2±75.2 | 1252.5±127.1 | 1252.7±77.6 | 1162.4±67.8 | 1536.8±95.5 | 1494.9±110.3 | 73.9±6.9 | 57.2±5.3 |
| **Σ 7-Monoene** | 115±8.6 | 151.3±12.3 | 271.5±25.5 | 430.6±32.3 | 138.6±12.9 | 142.1±13.8 | 52.4±2.8 | 49±4 | 269.3±23.3 | 153.1±14.2 | 852.8±66.6 | 734.2±50.2 |
| **Σ 5-Monoene** | 1.4±0.2 | 3.3±0.6 | 15.4±2 | 5.3±0.3 | 8.9±1.2 | 11.6±1.8 | 2.8±0.2 | 2.4±0.3 | 34.7±3.5 | 26.4±2.8 | 78.2±7.4 | 69.8±5 |
| **Total** | 1953.2±164.7 | 3347.9±262.8 | 3372.7±327.2 | 4000.6±259.7 | 2432.4±124.2 | 2613.8±215.5 | 1908.1±107.3 | 2105.3±102.6 | 2706.8±152.1 | 2604.2±173.2 | 1516.4±114.4 | 1444.6±94.7 |

**Table S3.** **Determining optimal cluster number^[[3]](#footnote-3)^ for K-means partition clustering analysis**.

| **Method** | **Optimal cluster number** | | | | | | | | |
| --- | --- | --- | --- | --- | --- | --- | --- | --- | --- |
|  | **3** | **5** | **9** | **4** | **8** | **2** | **10** | **6** | **7** |
| Ball | 3.95 | 1.17 | 0.46 | 1.81 | 0.58 | 15.94 | 0.41 | 0.88 | 0.70 |
| DB | 0.55 | 0.90 | 1.26 | 0.70 | 1.18 | 0.89 | 1.39 | 1.04 | 1.14 |
| Friedman | 634.80 | 728.44 | 874.73 | 701.46 | 852.93 | 249.14 | 883.22 | 821.86 | 831.69 |
| Hartigan | 170.99 | 28.56 | 5.04 | 65.12 | 33.79 | 460.75 | 0.93 | 20.57 | 13.86 |
| Ptbiserial | 0.85 | 0.65 | 0.46 | 0.76 | 0.49 | 0.59 | 0.44 | 0.56 | 0.51 |
| Ratkowsky | 0.44 | 0.37 | 0.29 | 0.40 | 0.30 | 0.37 | 0.28 | 0.34 | 0.32 |
| Scott | 2030.92 | 3052.91 | 4145.38 | 2618.86 | 3857.75 | 563.94 | 4237.62 | 3477.66 | 3695.24 |
| SDindex | 7.13 | 10.56 | 16.29 | 7.28 | 18.17 | 17.12 | 29.93 | 15.37 | 15.96 |
| Silhouette | 0.63 | 0.44 | 0.35 | 0.53 | 0.39 | 0.48 | 0.31 | 0.40 | 0.39 |
| TraceW | 11.84 | 5.85 | 4.14 | 7.26 | 4.67 | 31.89 | 4.06 | 5.29 | 4.91 |
| TrCovW | 1.61 | 0.20 | 0.09 | 0.37 | 0.12 | 19.03 | 0.08 | 0.16 | 0.13 |
| Beale | 2.15 | -7.83 | -5.75 | 7.32 | -4.17 | 58.82 | -2.71 | 1.64 | -10.75 |
| CCC | 19.12 | 25.83 | 21.31 | 23.66 | 20.26 | 2.53 | 18.26 | 24.63 | 22.54 |
| Duda | 0.89 | 1.77 | 1.48 | 0.71 | 1.31 | 0.24 | 1.18 | 0.92 | 2.54 |
| Pseudot2 | 21.52 | -48.45 | -20.80 | 37.97 | -13.19 | 624.92 | -6.77 | 6.62 | -49.10 |
| Dunn | 0.09 | 0.14 | 0.13 | 0.13 | 0.10 | 0.08 | 0.12 | 0.10 | 0.12 |
| Gap | 0.65 | 0.83 | 0.91 | 0.78 | 0.90 | 0.33 | 0.92 | 0.86 | 0.88 |
| KL | 4.32 | 2.64 | 10.93 | 3.30 | 0.39 | 1.26 | 2.84 | 1.51 | 1.62 |
| Rubin | 5.04 | 10.21 | 14.42 | 8.23 | 12.80 | 1.87 | 14.69 | 11.30 | 12.16 |
| CH | 548.06 | 619.52 | 444.55 | 650.50 | 448.21 | 237.36 | 401.72 | 551.89 | 496.79 |
| Cindex | 0.29 | 0.29 | 0.32 | 0.31 | 0.26 | 0.40 | 0.33 | 0.28 | 0.26 |
| McClain | 0.43 | 0.96 | 2.02 | 0.65 | 1.85 | 0.36 | 2.28 | 1.34 | 1.65 |
| SDbw | 0.16 | 0.07 | 0.05 | 0.09 | 0.05 | 0.50 | 0.05 | 0.06 | 0.06 |

**SUPPLEMENTAL FIGURES LEGNEDS**

Figure S1. **Representative GC-MS chromatogram traces of single 7-day-old virgin males and females from each species and strain.** Female CHCs are shown at the top in red, and male CHCs in mirror-image at the bottom in blue. Compounds corresponding to each numbered peak are listed in Table 1. Compounds that are shared between sexes bear the same number. Arrows indicate peaks that were not perfectly resolved, with the minor component shown by dashed lines. Unit-less abundances are direct measurements from the flame ionized detector. *ES-1* (n-Hexacosane) and *ES-2* (n-Triacotane) are external standards used to calculate the absolute amounts of each compound. The total absolute amounts were: (A) females (3373±327 ng; n = 24) and males (4001±260 ng; n = 24) for *D. prolongata* Bavi strain; (B) females (2432±124 ng; n = 22) and males (2614±216 ng; n = 24) for *D. carroli*; (C) females (1908±107 ng; n = 18) and males (2105±260 ng; n = 20) for *D. rhopaloa*; (D) females (2707±152 ng; n = 33) and males (2604±173 ng; n = 26) for *D. kurseongensis*; (E) females (1516±114 ng; n = 18) and males (1445±95 ng; n = 19) for *D. fuyamai*.

Figure S2. **Comparison of CHC profiles between the sexes of each species.** The percentage of each CHC extracted from 7-day-old virgin males and females is represented as mean (± 95% confidence interval). Vertical axis was square root transformed to display low-abundance CHCs.

Figure S3. **Species- and sex-specific variation in CHC profiles.** Similar to Figure 3, principal component analysis was performed after (A) log-contrast transformation and (B) inclusion of the non-CHC component, cVA. (C) Non-metric multidimensional scaling was performed using the Bray–Curtis dissimilarity metric. When projecting dissimilarity in two-dimensional space, the stress value (0.055) was calculated using the R package “vegan.”

Figure S4. **Comparison of the remaining CHCs among species and sexes.** Similar to Figure 4, boxplots show the percentage distribution of each CHC. CHCs are ordered according to their relative contribution to the first two principal components as shown in Figure 3B.

Figure S5. **K-means partition clustering analysis across all individuals of the focal study group.** (A) Pairwise Euclidean dissimilarity matrix between samples using Visual Assessment of cluster Tendency (VAT) algorithm. The dissimilarity values between individuals were color coded, with highly similar individual pairs represented in red. The clustering tendency was confirmed by sharply demarcated red square blocks along the diagonal. (B) External cluster validation of 3 pre-specified clusters (Table S3) by silhouette plot. Individuals with positive silhouette width indicated higher resemblance to individuals within the same cluster than neighboring cluster, and silhouette width equal to 1 indicated perfect cluster assignment. Average silhouette width (0.63) was indicated by the red dotted line.

1. Compounds detected were in either quantifiable amounts (+) or trace amounts (tr). [↑](#footnote-ref-1)
2. Compounds were quantified by mean absolute amounts (±SEM in ng) over sample size n and summarized across the chemical class defined in Table 1. Trace amount (tr) was detectable but not quantified. Relative amount (in %) of each compound, or compounds of a given class, is reflected by heatmap shading, with more abundant compounds in red and less abundant in white. [↑](#footnote-ref-2)
3. Based on a variety of methods implemented in the R package “NbClust” and “factoextra”, objective functions were optimized for different cluster numbers (from 2 to 10). Values were calculated/estimated indices, with the optima highlighted in yellow shade. Proposals suggesting 3 as the optimal number of clusters were adopted, according to the majority rule. [↑](#footnote-ref-3)
