# Supplementary material for: Evolution of sexually dimorphic pheromone profiles coincides with increased number of male‐specific chemosensory organs in Drosophila prolongata
Source: Ecol Evol. 2019 Nov 17;9(23):13608–18. doi: 10.1002/ece3.5819 (PMC6912897; doi:10.1002/ece3.5819)
Supplement: Supplementary file 2 [file ECE3-9-13608-s002.pdf]

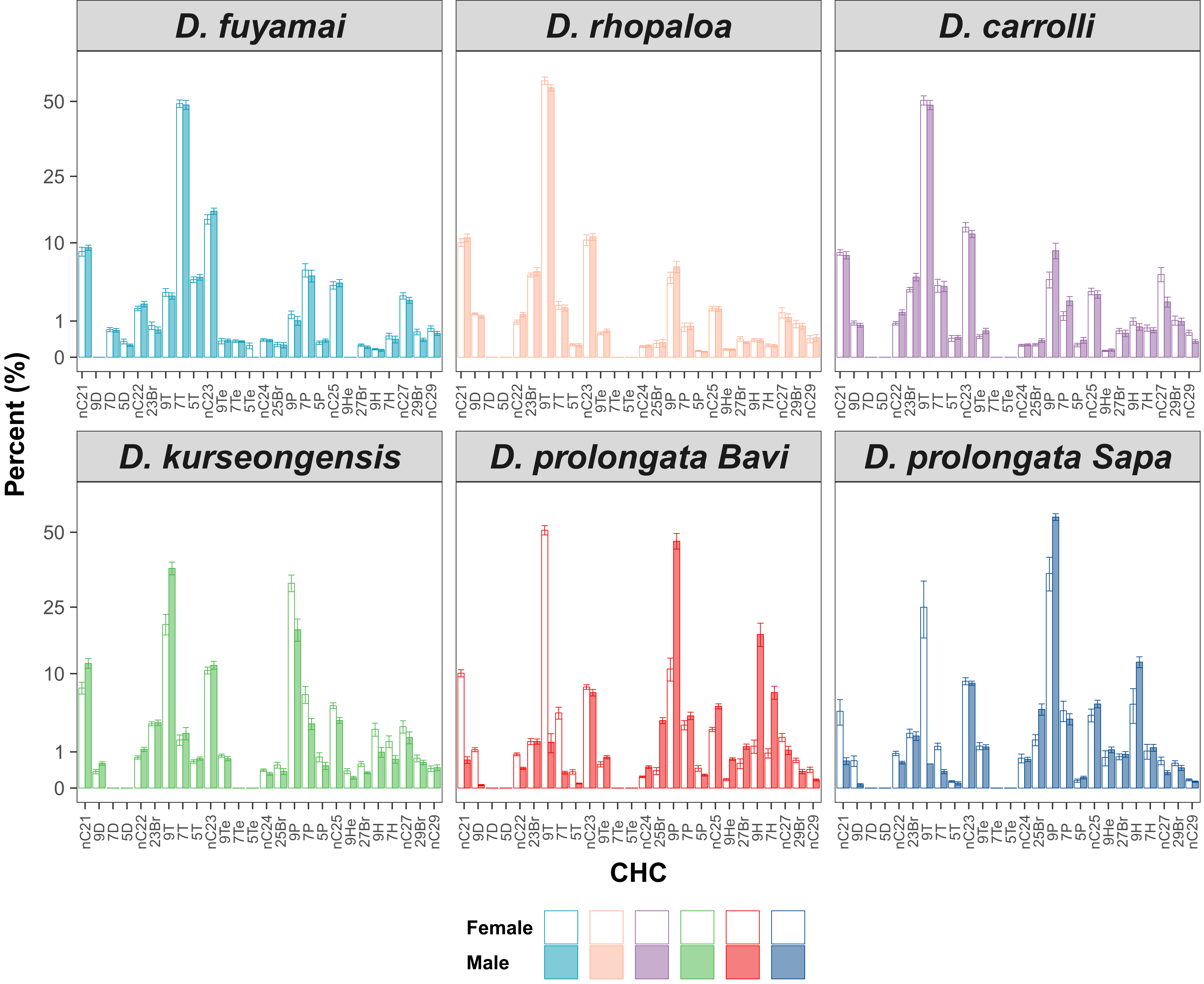

D. fuyamai
D. rhopaloa
D. carrolli
D. kurseongensis
D. prolongata Bavi
D. prolongata Sapa
Percent (%)
50
25
10
1
0
nC21 9D 7D 5D nC22 23Br 9T 7T 5T nC23 9Te 7Te 5Te nC24 25Br 9P 7P 5P nC25 9He 27Br 9H 7H nC27 29Br nC29
CHC
Female
Male
